# PARP1 and CHK1 coordinate PLK1 enzymatic activity during the DNA damage response to promote homologous recombination-mediated repair

**DOI:** 10.1093/nar/gkab584

**Published:** 2021-07-01

**Authors:** Bin Peng, Ruifeng Shi, Jing Bian, Yuwei Li, Peipei Wang, Hailong Wang, Ji Liao, Wei-Guo Zhu, Xingzhi Xu

**Affiliations:** Guangdong Key Laboratory for Genome Stability & Disease Prevention and Carson International Cancer Center, Marshall Laboratory of Biomedical Engineering, Shenzhen University School of Medicine, Shenzhen, Guangdong 518060, China; Guangdong Key Laboratory for Genome Stability & Disease Prevention and Carson International Cancer Center, Marshall Laboratory of Biomedical Engineering, Shenzhen University School of Medicine, Shenzhen, Guangdong 518060, China; Shenzhen University-Friedrich Schiller Universität Jena Joint PhD Program in Biomedical Sciences, Shenzhen University School of Medicine, Shenzhen, Guangdong 518060, China; Guangdong Key Laboratory for Genome Stability & Disease Prevention and Carson International Cancer Center, Marshall Laboratory of Biomedical Engineering, Shenzhen University School of Medicine, Shenzhen, Guangdong 518060, China; Guangdong Key Laboratory for Genome Stability & Disease Prevention and Carson International Cancer Center, Marshall Laboratory of Biomedical Engineering, Shenzhen University School of Medicine, Shenzhen, Guangdong 518060, China; Guangdong Key Laboratory for Genome Stability & Disease Prevention and Carson International Cancer Center, Marshall Laboratory of Biomedical Engineering, Shenzhen University School of Medicine, Shenzhen, Guangdong 518060, China; College of Life Sciences, Capital Normal University, Beijing 100048, China; Guangdong Key Laboratory for Genome Stability & Disease Prevention and Carson International Cancer Center, Marshall Laboratory of Biomedical Engineering, Shenzhen University School of Medicine, Shenzhen, Guangdong 518060, China; Guangdong Key Laboratory for Genome Stability & Disease Prevention and Carson International Cancer Center, Marshall Laboratory of Biomedical Engineering, Shenzhen University School of Medicine, Shenzhen, Guangdong 518060, China; Guangdong Key Laboratory for Genome Stability & Disease Prevention and Carson International Cancer Center, Marshall Laboratory of Biomedical Engineering, Shenzhen University School of Medicine, Shenzhen, Guangdong 518060, China; Shenzhen University-Friedrich Schiller Universität Jena Joint PhD Program in Biomedical Sciences, Shenzhen University School of Medicine, Shenzhen, Guangdong 518060, China

## Abstract

Polo-like kinase 1 (PLK1) is a master kinase that regulates cell cycle progression. How its enzymatic activity is regulated in response to DNA damage is not fully understood. We show that PLK1 is enriched at double strand breaks (DSBs) within seconds of UV laser irradiation in a PARP-1-dependent manner and then disperses within 10 min in a PARG-dependent manner. Poly(ADP-)ribose (PAR) chains directly bind to PLK1 *in vitro* and inhibit its enzymatic activity. CHK1-mediated PLK1 phosphorylation at S137 prevents its binding to PAR and recruitment to DSBs but ensures PLK1 phosphorylation at T210 and its enzymatic activity toward RAD51 at S14. This subsequent phosphorylation event at S14 primes RAD51 for CHK1-mediated phosphorylation at T309, which is essential for full RAD51 activation. This CHK1–PLK1–RAD51 axis ultimately promotes homologous recombination (HR)-mediated repair and ensures chromosome stability and cellular radiosensitivity. These findings provide biological insight for combined cancer therapy using inhibitors of PARG and CHK1.

## INTRODUCTION

The human genome is constantly challenged and damaged by various environmental and endogenous factors. A delicately orchestrated array of biochemical reactions has thus evolved to ensure the high-fidelity repair of damaged DNA. Among the different types of DNA damage, DNA single strand breaks (SSBs), DNA double strand breaks (DSBs) and replication fork collapse are very detrimental to genome integrity (1,2).

In response to SSBs and DSBs, PARP1 is almost immediately recruited to and binds to the lesions via polyanion chains of ADP-ribose (PAR) moieties. Such PARylation at DNA lesions promotes local chromatin relaxation due to its negative charge, and histone displacement (3). This highly negative charge also facilitates the recruitment of DNA damage signaling and repair factors, such as MRE11, via non-covalent interactions with PAR-binding modules (4). Only a few PAR-binding modules have been characterized (5), including PBZ, FHA, the BRCT domain, macro domain, and OB-fold domain (6). Among these PAR-binding modules, we previously identified the PAR-binding regulatory (PbR) motif within the amino-terminus of the key checkpoint kinase CHK1. This binding stimulates CHK1 activity at the stalled replication fork (7).

PAR that accumulates on DNA breaks is degraded within minutes; this effect is mainly executed by the poly(ADP-ribose) glycohydrolase, PARG. PARG contains a macro domain and possesses exo-glycohydrolysis and endo-glycohydrolysis activity to hydrolyze the PAR chain into free ADP-ribose residues (8). Both the timely and orderly generation of PAR by PARP-1 and degradation of PAR by PARG are thus required for a proper DNA damage response.

The major mitotic kinases PLK1, Aurora A and Aurora B, are inhibited in response to DNA damage via various mechanisms. For example, PARP-1-mediated PARylation on Aurora B inhibits its enzymatic activity during mitosis in response to oxidative damage (9). CHK1-mediated phosphorylation on Aurora A inhibits its enzymatic activity in response to DSBs at G2 phase (10). PLK1 is the prototype member of the polo-like kinase (PLK) family (11). Like the four other family members, PLK1 has an N-terminal catalytic kinase domain (KD) and two C-terminal polo-box domains (PBD). PLK1 phosphorylates various substrates to regulate many essential steps throughout mitosis and cytokinesis (12). Increasing evidence suggests that PLK1 also has important roles in the DNA damage response. For example, PLK1 activity is inhibited by adriamycin treatment in the G2 phase of the cell cycle. This inhibition may prevent CDC25C activation and trigger the G2/M checkpoint (13). Indeed, PLK1 phosphorylation on two critical regulatory sites, S137 and T210, is inhibited after DNA damage (14). On the other hand, PLK1 directly phosphorylates RAD51 at S14 and facilitates homologous recombination (HR)-mediated DNA repair. A transient increase in PLK1-mediated RAD51 S14 phosphorylation is observed 20–40 min after DNA damage. The subsequent RAD51 phosphorylation on T13 by CK2 kinase promotes NBS1 recruitment and HR repair (15,16). Despite these advances in understanding, it remains a stigma how PLK1 is coordinately inactivated and reactivated after DNA damage. Here, we aimed to address this knowledge gap by performing a series of *in vitro* and *in vivo* biochemical assays. We show that PLK1 is recruited to DSBs within seconds through PAR binding and removed from these damage sites within minutes through PAR degradation. Our delineation of the underlying mechanisms of this process might help further understand biological mechanism of synthetic lethality therapy involved PARP/PARG inhibitors.

## MATERIALS AND METHODS

### Cell culture, plasmid construction, reagents and antibodies

Human U2OS cells, the ER-*Asi*SI-expressing U2OS cells, DR-U2OS cells, *Parp1*^–/–^ MEF cells (17) (a kind gift from Dr Zhao-Qi Wang's lab), 293T, HeLa and *LIG4*^–/–^ HeLa cells (a kind gift from Dr Jun Huang's lab) were cultured at 37°C in a humidified incubator with 5% CO_2_ in DMEM (HyClone, SH30022.01) supplemented with 10% fetal bovine serum (PAN, ST30-3302) and 1% penicillin/streptomycin (HyClone, SV30010). MCF10A cells were cultured with DMEM/F12 (HyClone), EGF (20 ng/ml), hydrocortisone (0.5 mg/ml), insulin (10 μg/ml) supplemented with fetal bovine serum and penicillin/streptomycin.

PLK1, CHK1 and RAD51 cDNAs were sub-cloned into a pcDNA3.0-HA or pcDNA3.0-FLAG vector. PLK1-5 were cloned into an EGFP-C1 or EGFP-N1 expression vector (Clontech). RAD51 cDNA was sub-cloned into lenti-blast-vectors (Novobio). Point mutations in PLK1 and RAD51 were generated using a Mut Express II Fast Mutagenesis Kit V2 (Vazyme). Bacteria expressing HIS tagged PLK1, Aurora A and RAD51 were generated via the pET28a system (Invitrogen). Bacteria expressing GST-tagged PLK1, RAD51 (1–86 aa), or HIS-RAD51 and HIS-PLK1 were generated via the pGEX-4T-1 system (GE Healthcare) and pET28 (a) expression vector (Novagen), respectively.

BlasticidinS HCl (R210-01) was purchased from Invitrogen. Camptothecin (CPT, C9911), gallotannin (tannic acid, V900190), UCN-01 (539644), 4-hydroxytamoxifen (4-OHT) (H7904) and nocodazole (M1404) were purchased from Sigma. KU55933 (S1092), NU6027 (S7114), NU7026 (S2893), Olaparib (AZD2281, S1060), PDD00017273 (S8862) and MLN8054 (S1100) were purchased from Selleck. Poly (ADP-ribose) (PAR) Polymer (PAR, 4336-100–01) and biotin (terminal)-poly ADP-ribose (PAR) polymer (Biotin-PAR, 4339-100–02) were purchased from Trevigen. Recombinant human active protein GST-CHK1 (1630-KS-010) produced in insect cells was purchase from R&D Systems.

Mouse monoclonal anti-PAR (4335-MC-100) was purchased from Trevigen. Rabbit polyclonal antibodies used in this study, including anti-HA (A190-208A), anti-PARP1(A301-375A), anti-53BP1 (A300-272A), anti-Aurora A (A300-071A), anti-GAPDH (A300–643A) and anti-CHK1 (A300-161A, IP), were purchased from bethyl. Anti-MRE11 (ab12159), anti-pRad51 (T309) (ab111568) and anti-PARG (ab236403) were purchased from Abcam. Other mouse monoclonal antibodies, including anti-FLAG (F1804) and anti-β-actin (A5441) were purchased from Sigma. The anti-FLAG^®^ M2 Affinity Gel (A2220) was purchased from Sigma. Anti-GFP (sc-9996), anti-PLK1 (sc-17783) anti-RAD51 (H-92, sc-8349) and anti-CHK1 (G-4, sc-8408, WB) were purchased from Santa Cruz. Anti-HIS (D291-3) and anti-GST (M209-3) were purchased from MBL. Mouse anti-PLK1 (pT210) (558400) was purchased from BD Pharmingen. Anti-phospho-PLK1 (Ser137) (07-1348) was purchase from Merck. Anti-Histone H3 Rabbit (A2348) and anti-Lamin B1 (A1910) were purchased from ABclonal. Anti-γH2AX (05-636) and anti-PARP2 (clone 4G8, MABE18) were purchased from Millipore. Rabbit phospho-specific antibodies against RAD51 (Ser14) were generated and affinity purified by Beijing B&M Biotech using the phosphor peptide (NH2)-CEANADTpSVEEE-(COOH).

### Laser-microirradiation and ionizing radiation (IR)

U2OS and HeLa cells were grown on a dish with a thin glass bottom and then locally irradiated with a 365 nm pulsed nitrogen UV laser (16 Hz pulse, 55% laser output) generated from a Micropoint System (Andor). Images were captured in real time every 20 s under a DragonFly confocal imaging system (Andor). The fluorescence intensity was determined with ImageJ (NIH). For IR, the cells were exposed to a Radsource RS-2000pro X-Ray irradiator at a dose rate of 1.67 gray (Gy)/min.

### RNA interference

The following siRNA oligonucleotide duplexes were used:

RAD51 (3′UTR, 5′-GACUGCCAGGAUAAAGCUUdTdT-3′);

PARP1 (5′-CAAAGUAUCCCAAGAAGUUdTdT-3′);

PARP2 (5′-GGAGAAGGAUGGUGAGAAAdTdT-3′);

and PARG (5′-GGAUAAGGUACUUGAAGAAdTdT-3′) (18).

All siRNAs were transfected into cells using lipofectamine RNAiMAX for 48 h before harvest, according to the manufacturers conditions (Invitrogen).

### Immunoprecipitation, immunoblotting and immunofluorescence

Immunoprecipitation and immunoblotting were performed as previously described (19). For indirect immunofluorescence staining, U2OS cells were first micro-irradiated with a MicroPoint System (Andor) as described above, while HeLa cells were cultured on coverslips. Both cell lines were washed once with PBS, fixed with 4% paraformaldehyde (PFA) at room temperature for 5 min, permeabilized with Triton-X100 (0.5%) for 5 min, and then blocked with 2% BSA in 0.1% PBST for 30 min. The cells were incubated with primary antibody for 60 min, washed three times with PBST and incubated with a fluorescent-conjugated secondary antibody for 60 min. After extensive washing with PBST, the cells were counter-stained with DAPI for 2 min to label the nuclei. Images were captured under a DragonFly confocal imaging system (Andor).

### Chromatin fractionation

Chromatin fractionation was performed as previously described (19).

### GST pulldown assays and *in vitro* kinase assays

Bacterially-produced or insect cells-produced GST fusions protein (1 μg) were incubated with bacterially produced HIS tagged fusion proteins (1 μg) in 500 μl NETN buffer [20 mM Tris–HCl (pH 8.0), 0.15 M NaCl, 1 mM EDTA, 0.5% NP-40 and a protease inhibitor cocktail] at 4°C overnight. Glutathione-sepharose beads were added and incubated for 1 h before extensively washing with NETN buffer.

*In vitro* kinase assays were performed as previously described (20) except that the PLK1 kinase buffer contained 25 mM HEPES buffer (pH 7.4), 50 mM NaCl, 1 mM Na_3_VO4, 10 mM MgCl_2_,1 mM DTT, 10 μCi ^32^P-γ-ATP (Perkin Elmer) or 100 μM γ−ATP. The kinase reaction was carried out at 30°C for 30 min and then analyzed by SDS-PAGE, autoradiography or western blotting. The GST pulldown after the kinase assay was performed by adding 500 μl NETN buffer into the reaction after the *in vitro* kinase assay was completed.

### Generation of HeLa cells stably expressing wild-type or mutant FLAG-RAD51

The coding region of wild-type RAD51 or its point mutants was sub-cloned into lenti-blast-vectors. The lenti-virus constructs were co-transfected with psPAX2 and pMD2.G into 293T cells. The culture medium was collected 48 h after transfection, filtered through a micro-filter with a pore size of 0.45 μm and subsequently used to infect HeLa cells in the presence of polybrene. The infectants were cultured in the presence of blasticidin. The surviving cell population was used for subsequent experiments.

### HR-mediated DSB repair

HR-mediated DSB repair assays used DR-U2OS cells, in which a single copy of DR-GFP reporter gene has been integrated into its genome. The assays were performed as previously described (21). The collected cells were analyzed using a BD Accuri C6 flow cytometer.

For the I-SceI induced DSB assay, the DR-U2OS cells were infected with the I-SceI lenti-virus for 48 h and then analyzed by immunostainning with PLK1, γ-H2AX or 53BP1 antibodies.

### Poly (ADP-ribose) (PAR) binding assays

Approximately 1 μg of each recombinant protein was incubated with 20 pmol PAR in NETN buffer overnight at 4°C. Glutathione agarose was then added to the reaction mix and incubated for a further 1 h. The beads were then extensively washed with NETN buffer. The reaction was analyzed by dot-blotting onto PVDF membranes and immunoblotting with an anti-PAR antibody.

### ER-*Asi*SI system in U2OS

The ER-*Asi*SI retrovirus was generated in 293T cells that had been transfected with pBABE-ER-*Asi*SI, pCS2-mGP and pMD2G. The supernatant containing the retrovirus was collected, filtered through a 0.45 μm syringe filter, and then used to infect U2OS cells under puromycin selection. ER-*Asi*SI-expressing U2OS cells were treated with 4-hydroxytamoxifen (4-OHT) for 4 h to allow for *Asi*SI to enter the nucleus and generate DSBs.

### Chromatin immunoprecipitation assay

ER-*Asi*SI-expressing U2OS cells were treated with 4-OHT for 4 h. Chromatin immunoprecipitation (ChIP) assay was performed as previously described (22). Chromatin (200 μg) was immunoprecipitated with IgG, PLK1, MRE11 or FLAG antibodies (2 μg). The immunoprecipitated DNA and input DNA were analyzed by qPCR, using the following primers: DSB.F: 5′-GATTGGCTATGGGTGTGGAC-3′; DSB.R: 5′-CATCCTTGCAAACCAGTCCT-3′. The IP efficiency was calculated as the percent of the input DNA immunoprecipitated (23).

### Chromosome aberration assay

Briefly, the cells were exposed to IR (5 Gy) and then treated with colchicines (0.4 μg/ml) for 6 h before harvesting. The collected cells were incubated in hypotonic solution (75 mM KCl) for 30 min, and fixed in a 3:1 methanol/acetic acid solution (three washes) and stored overnight at −20°C. The cells were then dropped onto slides, incubated for 2 h at 60°C and Giemsa-stained. Images were captured under a confocal imaging system (Andor). More than 100 mitotic chromosomes were randomly analyzed. Other chromosome aberrations include dicentric, deletion and ring, while fusion aberrations include telomere fusion and physical connection between chromatids outside the centromere regions.

### Quantification and statistical analysis

Experiments were performed three times independently. All statistical analyses were performed in Microsoft Excel. A two-tailed non-paired Student's *t*-test was used to determine significant differences between two treatments. A *P* value <0.05 was considered statistically significant.

## RESULTS

### Polo-like kinases are recruited to UV laser-induced DNA damage stripes

Much data support a role for PLKs in DNA damage repair. PLK1 phosphorylates RAD51at S14, facilitating CK2-mediated RAD51 phosphorylation at T13. This event promotes RAD51 recruitment by NBS1 and HR-mediated DSB repair (15,24). PLK3-mediated CtIP phosphorylation facilitates a CtIP–BRCA1 interaction that initiates end resection and non-homologous end joining (NHEJ) in G1 (25). We showed that PLK1-mediated CtIP phosphorylation at S723 facilitates error-prone micro-homology end joining and inactivation of the G2/M checkpoint (26). Finally, others showed that GFP-PLK1 is recruited to UV laser-induced DNA damage stripes (27).

**Figure 1.**
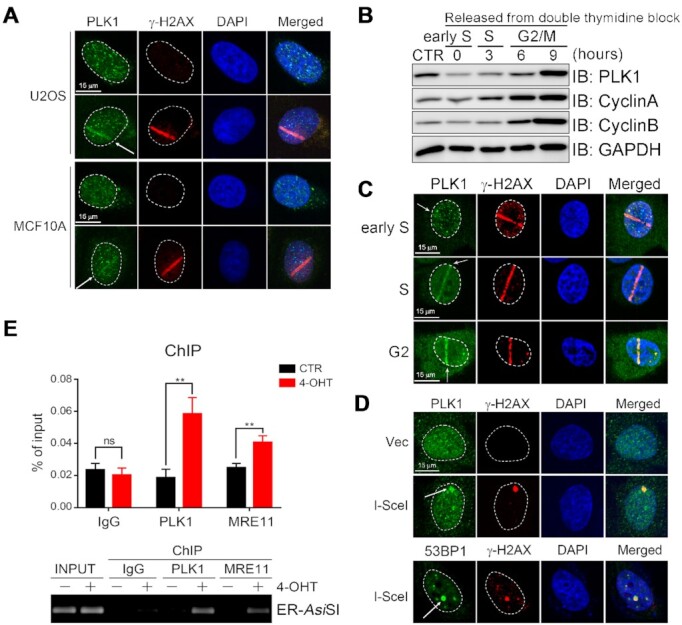
Endogenous PLK1 recruitment to UV laser-induced DNA damage stripes. (**A**) Endogenous PLK1 recruitment to DNA damage stripes. U2OS or MCF10A cells were treated or not with laser-microirradiated (arrows) before PLK1 and γ-H2AX analysis by immunofluorescence. (B and C) PLK1 expression and recruitment in different cell cycle stage. U2OS cells were synchronized at early S phase by double-thymidine block and then released for 0, 3, 6, 9 h, followed by western blotting with indicated antibody (**B**) and UV laser-microirradiation treatment (**C**) before PLK1 and γ-H2AX analysis by immunofluorescence. GAPDH used as a loading control, Cyclin A and Cyclin B were used as markers for S and the G2/M phase, respectively. (**D**) PLK1 recruitment to I-SceI-induced DSBs in DR-GFP cells. DR-GFP cells were infected with I-SceI lenti virus for 48 h, PLK1, γ-H2AX and 53BP1 were analyzed by immunofluorescence. (**E**) PLK1 enrichment at DSBs induced by 4-OHT. ChIP assay was performed in ER-*Asi*SI U2OS cells treated or not with 4-OHT (300 nM) for 4 h, using the indicated antibodies. ChIP efficiencies were measured by qPCR from *Asi*SI induced DSBs. The data are derived from three independent experiments and represent the means ± SD. **P < 0.001; ns: not significant.

Despite these findings, there is no direct evidence showing that endogenous PLKs are enriched at DNA lesions during the DNA damage response (DDR). We thus attempted to determine if endogenous PLK1 is enriched at DNA lesions, using three independent approaches. First, we subjected U2OS and MCF10A cells to UV laser-microirradiation and then performed immunofluorescence staining 5 min later to detect PLK1 and γ-H2AX. Endogenous PLK1 co-localized with γ-H2AX at the DNA damage stripes in both cell lines (Figure 1A). As PLK1 expression is cell cycle dependent which begins at late G1 then increases from S and G2 phases and peaks at mitosis, U2OS cells were synchronized at early S phase through double-thymidine block and then released to progress from S to G2/M, followed by UV laser-microirradiation. We found PLK1 recruitment was consistent with its expression during different cell cycle stages (Figure 1B and C). Moreover, Fucci (fluorescent ubiquitination-based cell cycle indicator) is able to visualize dynamics of cell cycle progression in living cells. Cdt1 fused with a red fluorescent protein (mKO2) accumulates only in the G1 phase, while Geminin fused with a green fluorescent protein (mAG1) accumulates during S/G2/M phases (Supplementary Figure S1A) (28). We found that PLK1 expression and enrichment at DNA lesions in HeLa/Fucci cells was weak in Cdt1 positive cells, while increased expression and recruitment was found in Geminin positive cells (Supplementary Figure S1B).

Second, we transiently expressed I-SceI in DR-U2OS cells in which a single I-SceI site was engineered in the genome (21). Again, immunofluorescence staining revealed a single PLK1 focus that colocalized with the γ-H2AX focus (Figure 1D). Third, we added 4-hydroxytamoxifen (4-OHT) to *Asi*SI-ER U2OS cells (22) that stably express the restriction enzyme *Asi*SI fused to a modified estrogen receptor ligand binding domain. Translocation of *Asi*SI from the cytosol to the nucleus induced by 4-OHT generates multiple, sequence-specific and unambiguously positioned DSBs across the genome. Chromatin immunoprecipitation (ChIP) assays uncovered that both endogenous and FLAG-tagged PLK1, like MRE11, was enriched at DSB sites (Figure 1E and Supplementary S1C).

To rule out the possibility of cross-reactivity of the PLK1 antibodies we used, we examined the recruitment dynamics of GFP-PLK1 and other GFP-tagged PLKs to the UV laser-induced DNA damage stripes. When we expressed GFP-PLKs in U2OS cells, both GFP-PLK1 and GFP-PLK2 were recruited to DNA damage stripes within 20 s after UV laser irradiation and peaked at 5 min. GFP-PLK3 was recruited 2 min after irradiation, and neither GFP-PLK4 nor GFP-PLK5 exhibited any obvious enrichment at DNA damage stripes after 10 min (Supplementary Figures S1D and S1E). To rule out the possibility of non-specific relocation due to amino-terminal tagging, we placed the GFP tag at the carboxyl termini of the PLKs (PLKs-GFP) and obtained comparable results (Supplementary Figure S1F).

Next, we explored which PLK domain is sufficient for enrichment at DNA damage stripes. Both the kinase domains (KDs) and polo-box domains (PBDs) of PLK1-3 were recruited to DNA lesions after UV laser-induced irradiation; however, the PBDs of PLK2 and PLK3 exhibited less extensive recruitment when compared to the KDs (Supplementary Figures S1G, S1H and S1I). Neither the KDs nor PBDs of PLK4 and PLK5 were recruited to DNA damage stripes (Supplementary Figure S1I and data not shown). Nucleotide binding domains (NBDs) are highly conserved through PLK1 to PLK3 (Supplementary Figure S1J). To verify whether NBD is essential for PLKs recruitment, depleting the first PLK1 nucleotide binding domain (59–67 aa), but not the second (178-191 aa), abolished PLK1 recruitment to DNA lesions (Supplementary Figures S1K and M). Deleting the first nucleotide binding domain in PLK2 or PLK3 diminished their enrichment at the DNA damage stripes (Supplementary Figures S1L and M). We substituted individual amino acid to alanine of NBD (59-LGKGGFAKC-67) in PLK1, however, the recruitment of these mutations was similar with WT (data not shown). These results suggest that the whole NBD of PLK1 was essential for its recruitment to DNA damage sites. These data demonstrate that GFP-tagged PLK1-3 (but not PLK4-5), and endogenous PLK1 can be recruited to UV laser-induced DNA damage stripes. The nucleotide binding domain is essential for this recruitment.

### PLK1 recruitment to and dissociation from DNA damage stripes is PARP-1-and PARG-dependent, respectively

Because PLK1 is the prototype member of the PLK family, we decided to pursue this kinase in depth in our subsequent investigations. We first attempted to determine if PLK1 enzymatic activity and PBD function are required for its recruitment. Both a kinase-dead mutant PLK1 (K82A) and a PBD-defective mutant PLK1 (W414F/H538A/K540A) (29), also known as PLK1 (FAA), were recruited to DNA damage stripes with similar dynamics to that of wild type PLK1 (Supplementary Figures S2A and S2B). These data suggest that neither enzymatic activity nor PBD function is important for its enrichment onto the damage sites.

**Figure 2.**
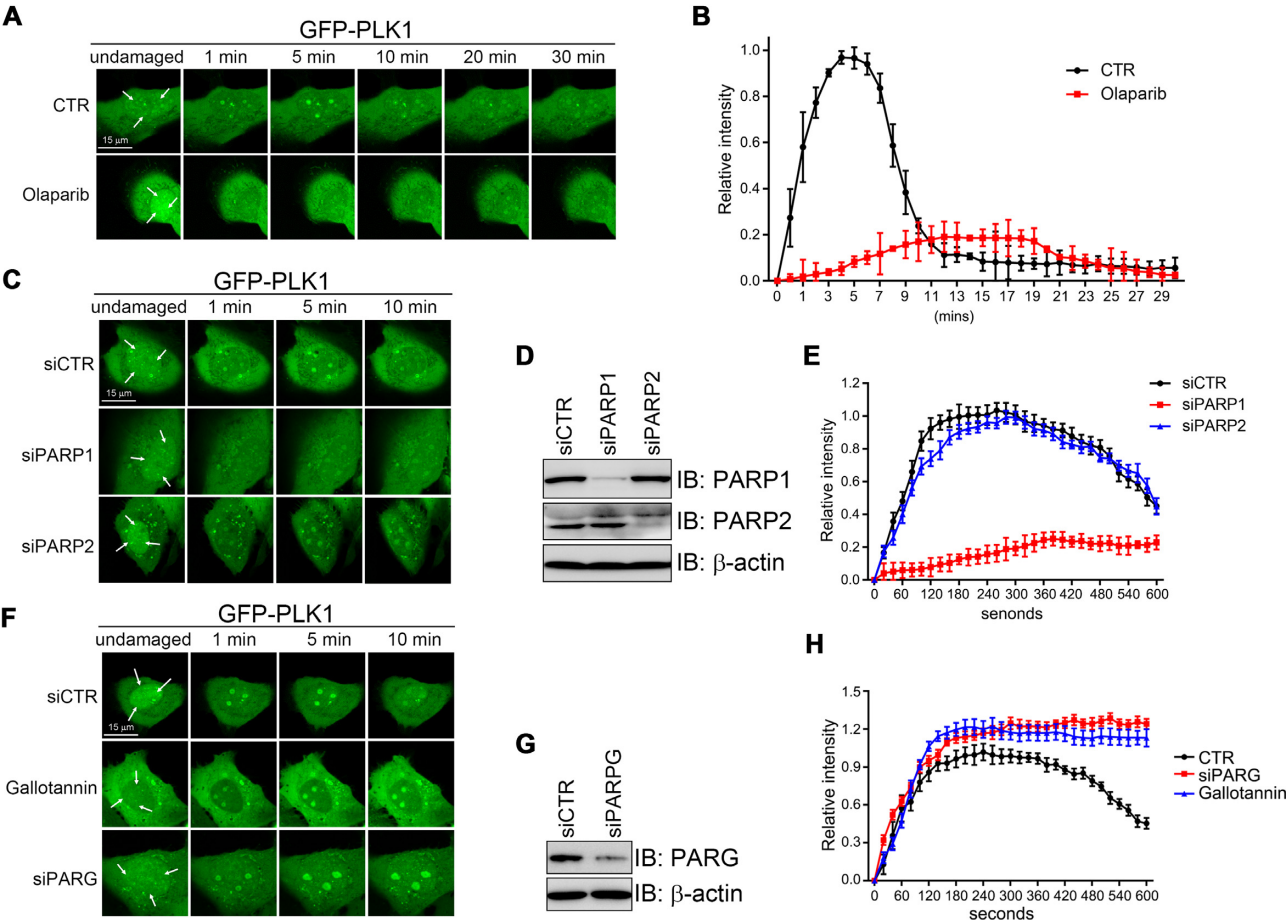
PLK1 recruitment to and dissociation from DNA damage stripes is PARP-1-and PARG-dependent, respectively. (A and B) Effects of olaparib (1 μM) on PLK1 recruitment to the DNA damage spots. U2OS cells transiently expressing GFP-PLK1 were laser-microirradiated at three spots (arrows). Representative time-lapse images (**A**) and the fluorescence intensity (**B**) of GFP-PLK1 at DNA damage spots are shown. (C–E) The role of PARP1 and PARP2 on PLK1 recruitment. U2OS cells were first transfected with control, PARP1 or PARP2 siRNAs for 24 h, followed by transfection with GFP-PLK1 for another 24 h before laser-microirradiation. The GFP-PLK1 recruitment (**C**), siRNA knockdown efficiency (**D**) and quantification of GFP-PLK1 fluorescence intensity at DNA damage spots (**E**) are shown. (F–H) The effect of PARG deficiency on PLK1 enrichment at DNA damage spots. U2OS cells were transfected with control or PARG siRNAs for 24 h, followed by transfection with GFP-PLK1 for another 24 h, and then subjected to laser-microirradiation. One set of transfectants was pretreated with the PARG inhibitor gallotannin (10 μM) for 2 h before microirradiation, The GFP-PLK1 recruitment (**F**), siRNA knockdown efficiency (**G**) and quantification of GFP-PLK1 fluorescence intensity at DNA damage spots (H) are shown. Data in (B), (E) and (H) are derived from three independent experiments.

To dissect PLK1 function in the DDR, we determined the recruitment kinetics of PLK1 to DNA damage spots induced by UV laser irradiation. We detected GFP-PLK1 at DNA damage spots in U2OS cells almost immediately after completing the micro-irradiation: the signal peaked at 5 min, and dispersed from the stripes 12 min after irradiation (Figure 2A and B). This acute recruitment suggests that PLK1 is an early DDR factor. We also determined the recruitment kinetics of endogenous and GFP-PLK1 to ER-*Asi*S1-restriction-enzyme-induced DSBs. Both endogenous and epitope-tagged PLK1 were enriched at DSBs sites, peaked at 2h then decrease at 4h in the presence of 4-OHT (Supplementary Figures S2C and D). The finding further supports that PLK1 is enriched at DSBs. We thus tend to think that PLK1 is recruited to UV laser-induced DNA lesions, though we could not rule out the possibility that PLK1 could be recruited to UV laser-induced single strand DNA breaks.

We then thought to determine the PLK1 upstream regulators by individually pre-treating the cells with ATM, ATR, DNA-PKcs and PARP1 inhibitors. The ATM, ATR, and DNA-PKcs inhibitors had no obvious impact on PLK1 recruitment dynamics (Supplementary Figure S2E). However, pretreatment with the PARP1 inhibitor Olaparib diminished PLK1 recruitment (Figure 2A and B). Olaparib inhibits PARylation with a preference order of PARP1>PARP2>PARP3 (30), but only PARP1 and PARP2 have well-documented PARylation activity (31). We thus set out to determine if PARP1 and/or PARP2 regulate PLK1 recruitment. siRNA-mediated PARP1, but not PARP2 down regulation (Figure 2C and D), abolished PLK1 recruitment (Figure 2D and E). GFP-PLK1 recruitment was also abolished in *Parp1-/-* mouse embryonic fibroblasts (MEFs) (17) (Supplementary Figures S2F and S2G). These data suggest that PLK1 recruitment to DNA damage stripes is PARP1-dependent.

PLK1 retention at the DNA damage stripes only lasts for up to 12 min (Figure 2A and B). Coincidentally, PAR chains at DNA lesions are hydrolyzed by PARG within minutes (32). Given that PLK1 recruitment to DNA damage sites is PARP-1-dependent, we reasoned that PARG could drive PLK1 dispersal from the DNA damage sites. Indeed, inhibiting PARG expression by siRNA in U2OS cells (Figure 2F and G) resulted in sustained PLK1 retention at the DNA damage sites (Figure 2H). Similarly, treating U2OS cells with gallotannin, a cell-permeable PARG inhibitor that suppresses PAR hydrolysis (33,34), prolonged PLK1 retention at DNA lesions, but to a lesser extent than that in PARG-depleted cells (Figure 2F and H). When we pretreated the cells with a PARP1 inhibitor, observed no obvious endogenous PLK1 recruitment to the DNA damage stripes 5 min after UV laser irradiation (Supplementary Figure S2H). However, PARG inhibition by gallotannin or PDD00017273 had no effect on initial enrichment of both endogenous PLK1 and GFP-PLK1at, however, delayed its dissociation from DNA lesions induced by UV laser irradiation (Figure 2H, Supplementary Figures S2I and J), suggesting that PLK1 removal from the damaged sites is PARG-dependent. These results demonstrate that PLK1 recruitment to and dissociation from the DNA lesions is PARP1-dependent and PARG-dependent, respectively.

### PAR directly binds to PLK1 and inhibits its enzymatic activity

**Figure 3.**
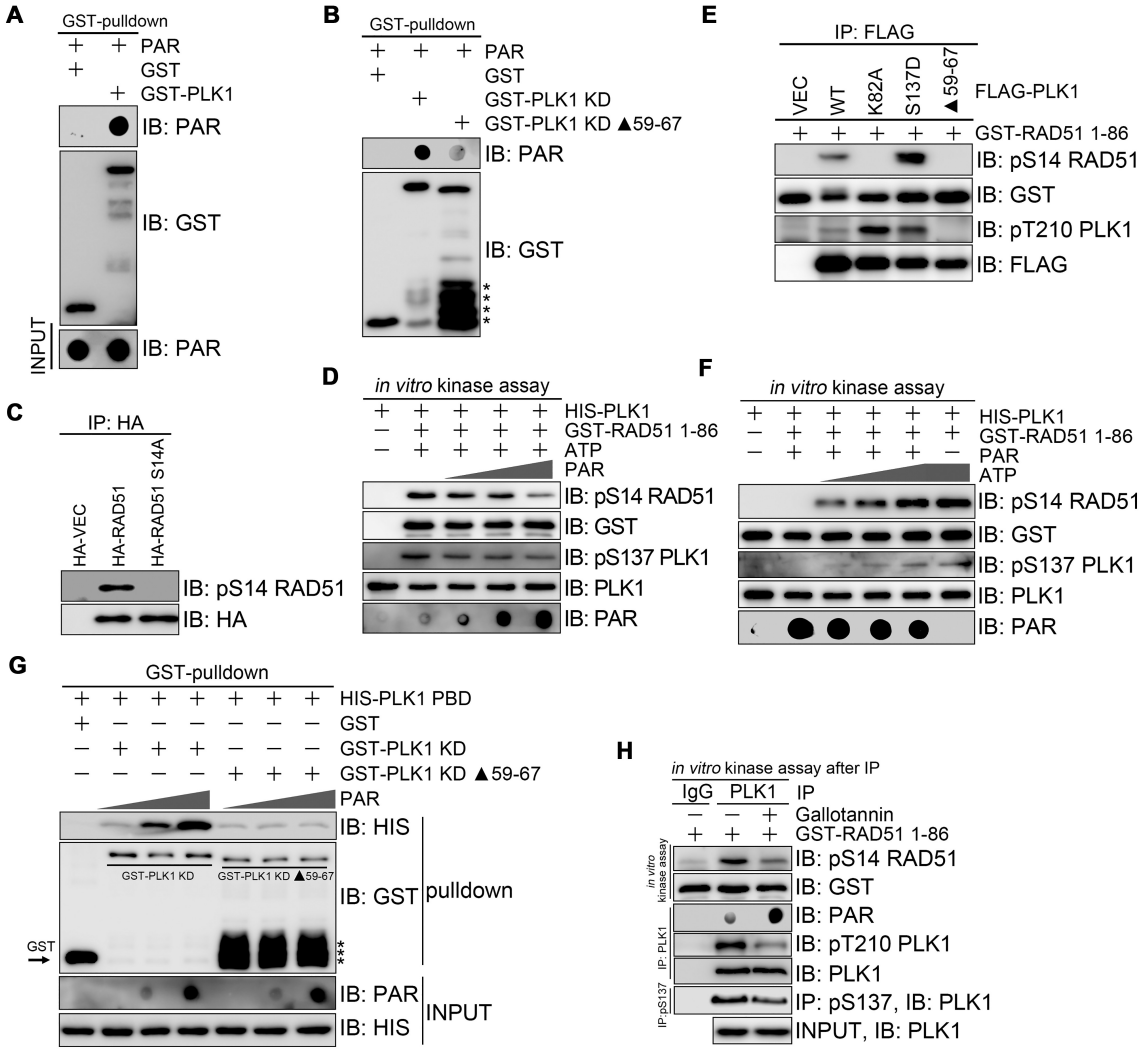
PAR directly binds to PLK1 and inhibits its enzymatic activity. (**A**) PLK1 binding to PAR. GST-PLK1 was used to pull down PAR (20 pmol) *in vitro*. (**B**) The role of the first PLK1 nucleotide binding domain in PAR binding. Kinase domain (KD) and a first nucleotide binding domain deletion mutant GST-PLK1KD Δ59–67 were used to pull down PAR (20 pmol) *in vitro*. *, non-specific signal. (**C**) The immunoreactivity of the phospho-specific-antibody against RAD51 Ser14. Total 293T cell lysates transiently expressing HA-VEC, HA-RAD51 or HA-RAD51 (S14A) were immunoprecipitated with HA before immunoblotting. (**D**) Effects of PAR binding on PLK1 enzymatic activity. GST-PLK1 was incubated with increasing amounts of PAR (0, 20, 80 pmol) before incubation with GST-RAD51 and γ-ATP. (**E**) Deletion of PAR binding domain abolished PLK1 enzymatic activity towards RAD51. Total cell lysates from 293T cells transiently expressing FLAG-VEC, FLAG-PLK1 or its mutants were immunoprecipitated with anti-FLAG. The resulting immunocomplexes were used for *in vitro* kinase assays with GST-RAD51(1-86). (**F**) Effects of ATP concentrations on PLK1 enzymatic activity in the presence of PAR. HIS-PLK1 saturately bound with PAR was used for *in vitro* kinase assays with GST-RAD51(1-86) and increasing concentrations of γ-ATP (0, 40, 100, 400 μM). (**G**) The effect of the PAR polymer on the interaction between PLK1 KD and PBD. Bacterially produced HIS-PLK1 PBD was pulled down by GST or GST-PLK1 KD or GST-PLK1 KD (Δ59–67) in the presence of increasing amounts of PAR (0, 20, 80 pmol) followed by immunoblotting with antibodies as indicated. *: degraded products of GST-PLK1 KD (Δ59–67). (**H**) The effect of PARG inhibition on PLK1 enzymatic activity *in vivo*. Total 293T cell lysates either mock-treated or pretreated with gallotannin (10 μM) were immunoprecipitated with pS137 PLK1 or PLK1 antibodies before *in vitro* kinase assay using GST-RAD51 as a substrate.

We next wanted to explore if PLK1 binds to PAR polymers. To do so, we co-incubated bacterially produced recombinant GST-PLK1 with PAR polymers, and performed GST pulldowns followed by dot blot analyses. PAR polymers were present in the GST-PLK1 pulldown complex, but not in the negative control (GST alone) (Figure 3A). Given that the first nucleotide binding domain is essential for PLK1 recruitment to DNA damage sites (Supplementary Figure S1K), we repeated our analyses using a GST-PLK1-KD or a deletion mutant of the first nucleotide binding domain GST-PLK1-KD (Δ59–67). Here, PLK1-KD but not the deletion mutant, bound to PAR polymers (Figure 3B). PLK1 thus directly binds to PAR polymers via the first nucleotide binding domain.

As PLK1 enzymatic activity is suppressed upon DNA damage (13,35), we speculated that PAR binding to PLK1 could inhibit its enzymatic activity. We generated a rabbit polyclonal phospho-specific antibody for RAD51 (S14) [here after known as pS14 RAD51], which was specifically reactive to HA-RAD51 but not HA-RAD51 (S14A) (Figure 3C). Then, we pre-incubated bacterially produced recombinant GST-PLK1 with increasing concentrations of PAR polymers and performed an *in vitro* kinase assay by adding ATP and recombinant GST-RAD51 (1–86 aa) (15). Immunoblotting with our pS14 RAD51 antibody revealed that pre-incubating PLK1 with PAR polymers inhibited its phosphorylation on S137 and enzymatic activity toward RAD51 in a PAR-polymer dose-dependent manner (Figure 3D). Deletion of the PAR binding domain in PLK1 resulted in loss of its enzymatic activity and failed to phosphorylate RAD51 (Figure 3E). Similar *in vitro* kinase assay was performed using PAR-saturated HIS-PLK1 and increasing concentrations of ATP. It was found that high concentration of ATP led to PLK1 activation, as evidenced by pS137 PLK1 and pS14 RAD51 (Figure 3F), indicating ATP might compete with PAR for the nucleotide binding domain of PLK1.

Intramolecular binding of the PLK1 PBD to its kinase domain inhibits PLK1 enzymatic activity (36). We thus wanted to determine if PAR binding to PLK1 impacts on PBD–KD binding. We again pre-incubated GST-PLK1 KD or GST-PLK1 KD (Δ59–67) with increasing concentrations of PAR polymers and then further incubated the reaction with recombinant HIS-PLK1 PBD. Here, binding between PLK1-KD, but not PLK1 KD (Δ59–67), and PLK1-PBD increased in a PAR-polymer dose-dependent manner (Figure 3G).

If PAR binding to PLK1 inhibits its enzymatic activity, PAR accumulation *in vivo* should inhibit PLK1 enzymatic activity. Indeed, when we pretreated the cells with the PARG inhibitor gallotannin, phosphorylation at S137 and T210 in the endogenous PLK1 immunoprecipitated complex and subsequent enzymatic activity toward RAD51 was reduced (Figure 3H). These findings demonstrate that PLK1 binding to PAR polymers inhibits its enzymatic activity possibly by promoting intramolecular binding between the PLK1 KD and the PBD (Supplementary Figure S3).

### CHK1 phosphorylates PLK1 at S137

**Figure 4.**
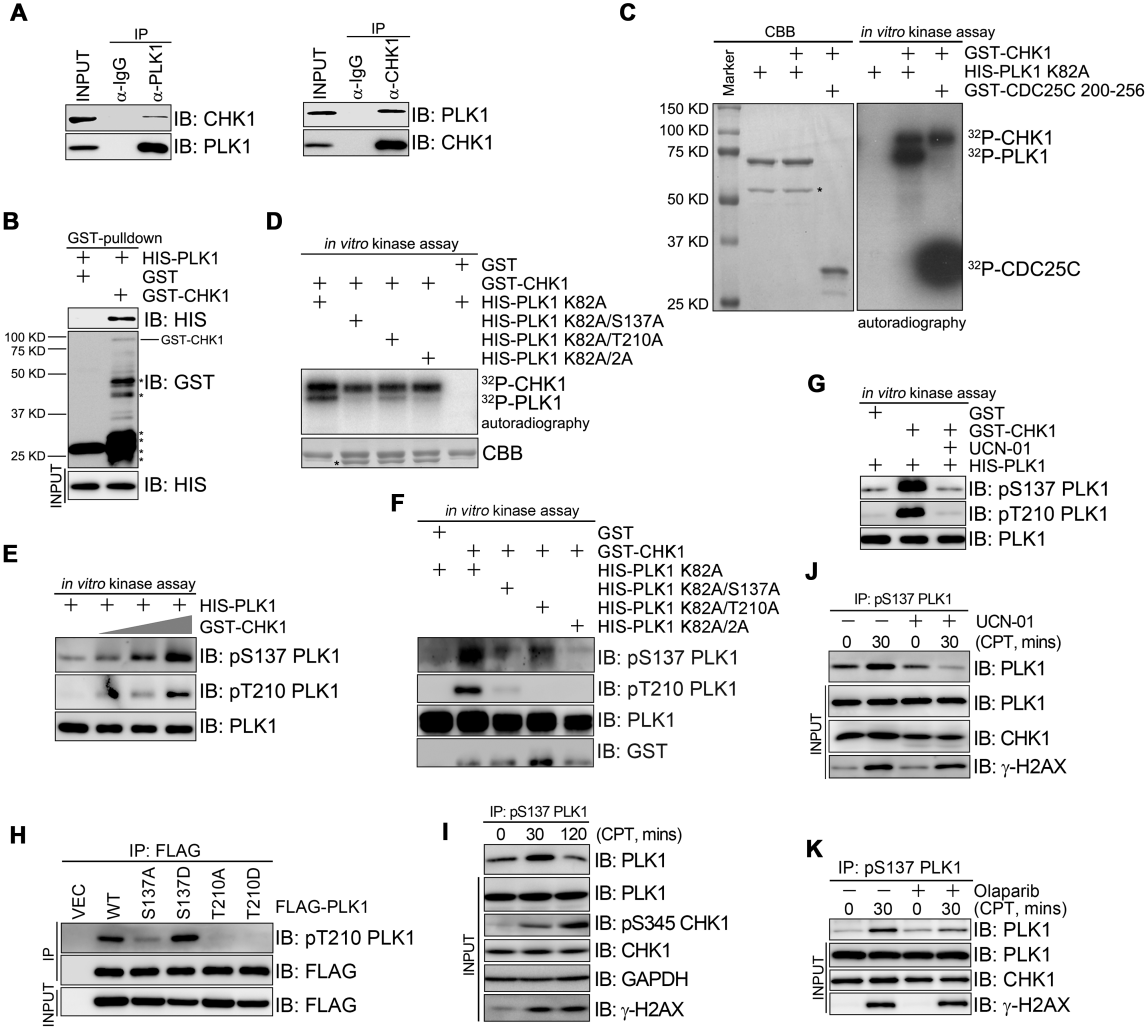
CHK1 phosphorylates PLK1 at S137. (**A**) PLK1 interaction with CHK1. Total 293T cell lysates were immunoprecipitated with CHK1 or PLK1 antibodies before immunoblotting. (**B**) CHK1 interaction with PLK1. HIS-PLK1 was pulled down by GST or GST-CHK1 *in vitro* before immunoblotting. *, non-specific signal. (**C**) CHK1-mediated PLK1 phosphorylation *in vitro*. GST-CHK1 was incubated with HIS-PLK1 (K82A) or CDC25C (200–256) in the presence of ^32^P-γ-ATP. CHK1, PLK1 and CDC25C phosphorylation was detected by autoradiography. The gels were stained with CBB. *, non-specific signal. (**D**) CHK1-mediated PLK1 phosphorylation at S137 and T210. The indicated peptides were incubated with GST-CHK1 and ^32^P-γ-ATP before autoradiography. *, non-specific signal. (**E**) CHK1-mediated PLK1 phosphorylation in a dose dependent manner. An *in vitro* kinase assay was performed as described in C with increasing amounts of GST-CHK1 in the presence of cold γ-ATP. (**F**) The relationship between S137 and T210 phosphorylation. The indicated peptides were incubated with GST-CHK1 in the presence of γ-ATP, followed by immunoblotting. (**G**) Preferential PLK1 phosphorylation sites targeted by CHK1 *in vitro*. An *in vitro* kinase assay was performed as described in C, in the presence or not of the UCN-01 inhibitor (50 nM). (**H**) Preferential PLK1 phosphorylation sites targeted by CHK1 *in vivo*. Total 293T cell lysates transiently expressing the indicated constructs were immunoprecipitated with a FLAG antibody before immunoblotting. (**I**) PLK1 phosphorylation dynamics at S137 after DNA damage. Total 293T cell lysates treated with CPT (2 μM) for 30 min were immunoprecipitated with pS137 PLK1 before immunoblotting. (**J** and **K**) DNA damage-induced PLK1 phosphorylation at S137 after UCN-01 or Olaparib treatment. 293T cells were pre-treated with UCN-01 (10 nM) or Olaparib for 60 min were immunoprecipitated with pS137 PLK1 before immunoblotting.

PLK1 is activated at an early timepoint during S/G2 phase in response to DNA damage; this event contributes to HR-mediated DSB repair (15). How PLK1 is activated, however, is unknown. Our unpublished mass spectrometric analysis found that CHK1 was present in the endogenous PLK1 immunoprecipitation complex. Further co-immunoprecipitation assays confirmed that both endogenous and epitope-tagged PLK1 interacted with CHK1 (Figure 4A and Supplementary Figure S4A). A CHK1 construct (1-265) encompassing its kinase domain was sufficient to co-immunoprecipitate with PLK1 (Supplementary Figure S4B), while both the PLK1 KD and PBD could bind with CHK1, albeit the KD to a lesser extent (Supplementary Figure S4C). Mutating essential residues (W414A, H538A and K540A) in the PBD (29) compromised the PLK1–CHK1 interaction (Supplementary Figure S4C).

The PBD serves as a phosphopeptide-binding module that preferentially recognizes the Ser-(pThr/pSer)-(Pro/X) motif (37). We thus hypothesized that the PLK1–CHK1 interaction could be induced by phosphorylation. Indeed, GSP-PLK1 PBD could pull down CHK1 in total lysate but failed to do so in a cell lysate pretreated with calf intestinal alkaline phosphatase (Supplementary Figure S4D). We then explored if PLK1 could serve as a substrate for CHK1. GST pull-down assays revealed that GST-CHK1 could pull down HIS-PLK1 (Figure 4B), indicating that PLK1 directly interacts with CHK1. By performing radiolabeled *in vitro* kinase assays, we uncovered that a CDC25C fragment (200–256 aa) encompassing the known S216 phosphorylation site integrated with the ^32^P signal. Similarly, kinase-dead PLK1 (K82A) was also radiolabeled by CHK1 (Figure 4C). To identify the phosphorylation sites mediated by CHK1, we analyzed *in vitro* phosphorylated HIS-PLK1 (K82A) in the presence of cold γATP by mass spectrometry. Both S137 and T210—critical regulatory phosphorylation sites in PLK1—were potential, high-confidence sites for phosphorylation by CHK1 (data not shown). This finding was supported by additional radiolabeled *in vitro* kinase assays using HIS-PLK1 (K82A/S137A), HIS-PLK1 (K82A/T210A), and HIS-PLK1 (K82A/S137A/T210A) (also known as HIS-PLK1(K82A/2A) as CHK1 substrates (Figure 4D). Here, PLK1 (K82A/S137A) and PLK1 (K82A/2A) showed a much lower radiolabel signal compared to that of PLK1 (K82A), while PLK1 (K82A/T210A) showed only a moderately lower radiolabel signal. These results suggest that both PLK1 S137 and T210 sites can be phosphorylated *in vitro* by CHK1, but that S137 is the major site. We verified these findings by performing *in vitro* kinase assays using phospho-specific antibodies against PLK1 S137 and T210 (Figure 4E-F). Preferential PLK1 phosphorylation at S137 by CHK1 could be blocked with CHK1 inhibitor UCN-01 (Figure 4G). Furthermore, immunoprecipitation (IP)-kinase assays revealed that a FLAG-PLK1 (S137A) immune complex from 293T cells was only weakly phosphorylated at T210, whereas FLAG-PLK1 (S137D) was highly phosphorylated at T210, to an equivalent level as seen with wild type FLAG-PLK1 (Figure 4H).

We then evaluated PLK1 phosphorylation at S137 *in vivo* using our pS137 PLK1 antibody. This antibody was suitable for immunoprecipitating endogenous PLK1 (Supplementary Figure S4E) and FLAG-PLK1 (Supplementary Figure S4F) from total cell lysates in nocodazole-treated cells but was not suitable for immunoblotting *in vivo*. Given this caveat, we used this phospho-specific antibody to enrich for pS137 PLK1 through immunoprecipitation for detection by immunoblotting using a regular PLK1 antibody. CPT treatment induced an increase in PLK1 S137 phosphorylation that peaked at 30 min; the levels returned to baseline 120 min after treatment (Figure 4I). Pre-treating the cells with the CHK1 inhibitor UCN-01 (Figure 4J) or PARP inhibitor Olaparib (Figure 4K) before CPT treatment prevented this increase in PLK1 phosphorylation (Figure 4J). We observed similar pS137 PLK1 dynamics when inducing DNA damage with 10 Gy irradiation (Supplementary Figures S4G and S4H). These results indicate that DNA damage-induced PLK1 activation depends on the formation of the PAR network.

Altogether, CHK1 phosphorylates PLK1 *in vitro* and *in vivo*. The major PLK1 phosphorylation site is S137, which is a prerequisite for further phosphorylation at T210 by CHK1.

### PLK1 (S137) phosphorylation blocks its binding to PAR and recruitment to the DNA damage sites

**Figure 5.**
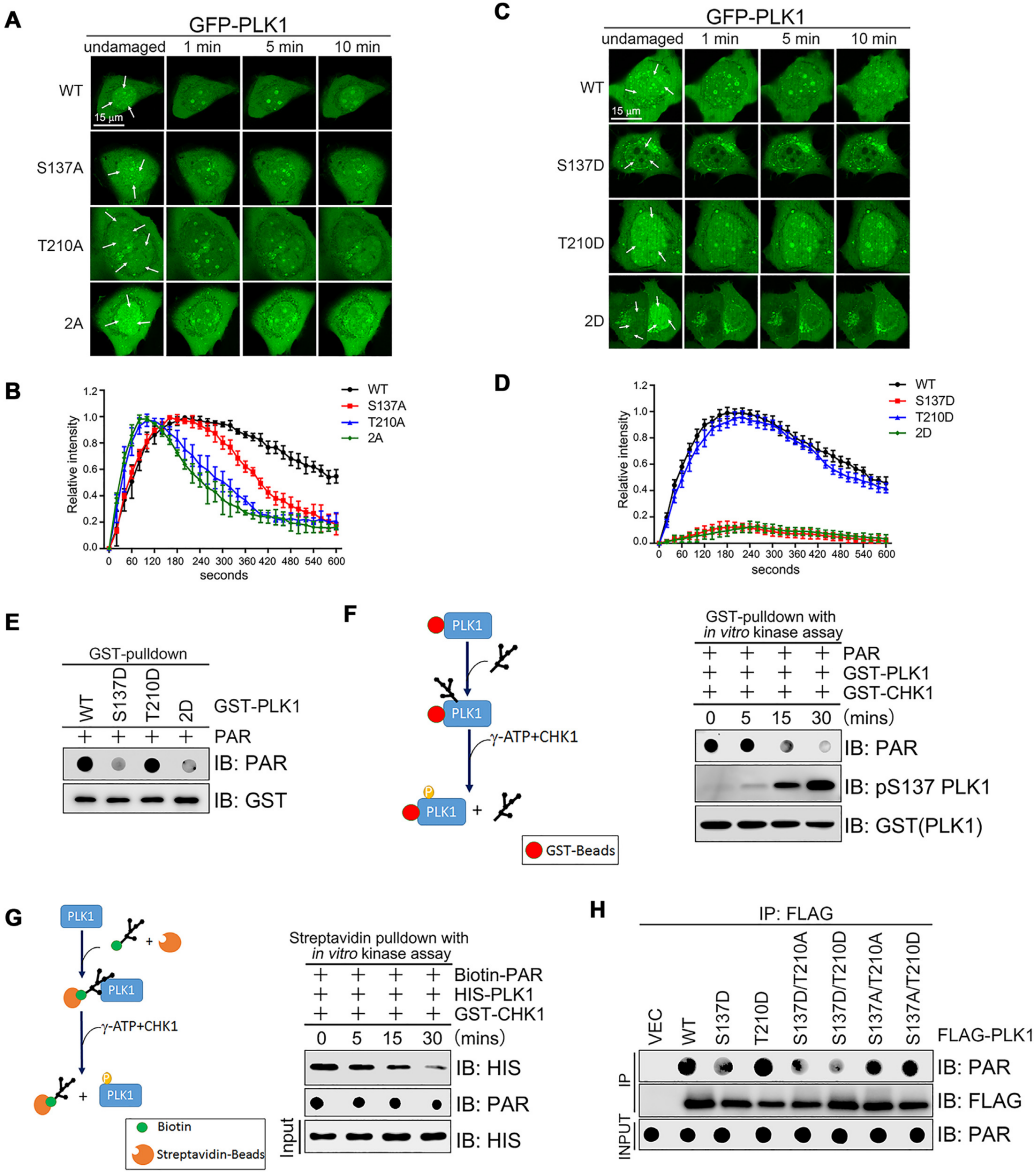
CHK1-mediated PLK1 (S137) phosphorylation blocks PLK1 recruitment to DNA damage sites and binding to PAR. (A, B) The role of T210 phosphorylation on PLK1 transient retention at DNA damage sites. U2OS cells transiently expressing the indicated constructs were laser-microirradiated at three spots (arrows). Representative time-lapse images (**A**) and the quantification of the GFP-PLK1 fluorescence intensity at DNA damage spots (**B**) are shown. (**C, D**) The effect of S137 phosphorylation on PLK1 recruitment to DNA damage sites. U2OS cells transiently expressing the indicated constructs were laser-microirradiated at three spots (arrows) and analyzed as described in A-B. Data in (B) and (D) are derived from three independent experiments. (**E**) The effect of a phosphorylation mimic mutant PLK1 (S137D) on PAR binding. PAR (20 pmol) was incubated with the indicated peptides before GST-pulldown and dot immunoblotting. (**F**) The effect of CHK1-mediated PLK1 phosphorylation at S137 on the PLK1–PAR complex. Schematic of the GST-pulldown (left). GST-PLK1 was immobilized on glutathione sepharose, incubated with PAR (20 pmol) and analyzed by *in vitro* kinase assay by incubating with CHK1 in the presence of γ-ATP (right). (**G**) The effect of PAR binding to PLK1 upon phosphorylation by CHK1. Schematic of the Streptavidin-pulldown (left). PLK1 was incubated with biotin-PAR (20 pmol), pulled down with streptavidin-sepharose beads, and analyzed by *in vitro* kinase assay by incubating with CHK1 in the presence of γ-ATP (right). (**H**) The effect of phosphorylation mimic PLK1 mutants at S137 on PAR binding. Total 293T cell lysates transiently expressing the indicated constructs were immunoprecipitated with a FLAG antibody, incubated with PAR (20 pmol) and then analyzed by immunoblotting/dot blotting.

Although we found that CHK1 mediates PLK1 phosphorylation at S137 and T210, we did not yet know the impact on PLK1 recruitment to DNA damage stripes. To test this, we engineered U2OS cells to express GFP- tagged PLK1 phosphorylation-defective mutants S137A, T210A, and S137A/T210A [denoted as GFP-PLK1 (2A)], or phosphorylation-mimic mutants S137D, T210D and 2D. After UV laser irradiation, GFP-PLK1 (S137A) exhibited similar recruitment dynamics to DNA damage sites as GFP-PLK1, but the retention time was shorter (Figure 5A, B and Supplementary Figure S5A). GFP-PLK1 (T210A) and the double mutant GFP-PLK1 (2A) showed the fastest initial recruitment to and removal from DNA lesions of all the PLK1 alleles (Figure 5A and B). Consistently, two of the phosphorylation-mimic mutants GFP-PLK1 (S137D) and GFP-PLK1 (2D) failed to be recruited to the DNA damage sites, supporting that PLK1 (S137) phosphorylation prevent its recruitment to the DNA damage site. Meanwhile, GFP-PLK1 (T210D) exhibited an almost identical recruitment pattern to GFP-PLK1 (Figure 5C, D and Supplementary Figure S5B). We also observed that GFP-PLK1 (S137A/T210D) displayed similar recruitment dynamics to GFP-PLK1 (S137A), while GFP-PLK1 (S137D/T210A) could not be recruited to the DNA damage stripes (Supplementary Figure S5C). These findings indicate that unphosphorylated PLK1 at S137 favors the initial recruitment of PLK1 to DNA lesions, while PLK1 phosphorylation at S137 disfavors initial recruitment no matter the phosphorylation status of T210. PLK1 phosphorylation at T210 ensures its retention at the DNA lesion only when S137 is not phosphorylated. Given that both the S137D and T210D mutations increase PLK1 enzymatic activity (38), and that PLK1 enzymatic activity is not important for its recruitment to the DNA damage site (Supplementary Figure S2A), we reasoned that CHK1-mediated PLK1 phosphorylation at S137 might prevent PAR binding. To test our hypothesis, we examined the binding capacity of PLK1 and its phosphorylation-mimic mutants to PAR *in vitro* by GST pulldown assay. We found that while both GST-PLK1 and GST-PLK1 (T210D) bound to PAR, GST-PLK1 (S137D) or GST-PLK1 (2D) only bound weakly to PAR (Figure 5E).

Next, we incubated GST-PLK1 with an excess of PAR for 1 h, then added CHK1 and γ−ATP for different durations. Here, PLK1 phosphorylation at S137 increased in a time-dependent manner, whereas PLK1–PAR binding decreased accordingly (Figure 5F). Reciprocally, streptavidin pulldown assays showed that under similar conditions, biotin-PAR pulled down less PLK1 in a time-dependent manner (Figure 5G). Furthermore, FLAG immunocomplexes with FLAG-PLK1 (T210D), FLAG-PLK1 (S137A/T210A), or FLAG-PLK1 (S137A/T210D) bound to a similar amount of PAR as FLAG-PLK1, whereas FLAG immunocomplexes with FLAG-PLK1 (S137D), FLAG-PLK1 (S137D/T210A), or FLAG-PLK1 (S137D/T210D) exhibited a low level of PAR binding (Figure 5H). These results demonstrate that CHK1-mediated PLK1 S137 phosphorylation might promote its dissociation with PAR.

### CHK1-mediated PLK1 (S137) phosphorylation promotes PLK1 enzymatic activity toward RAD51

**Figure 6.**
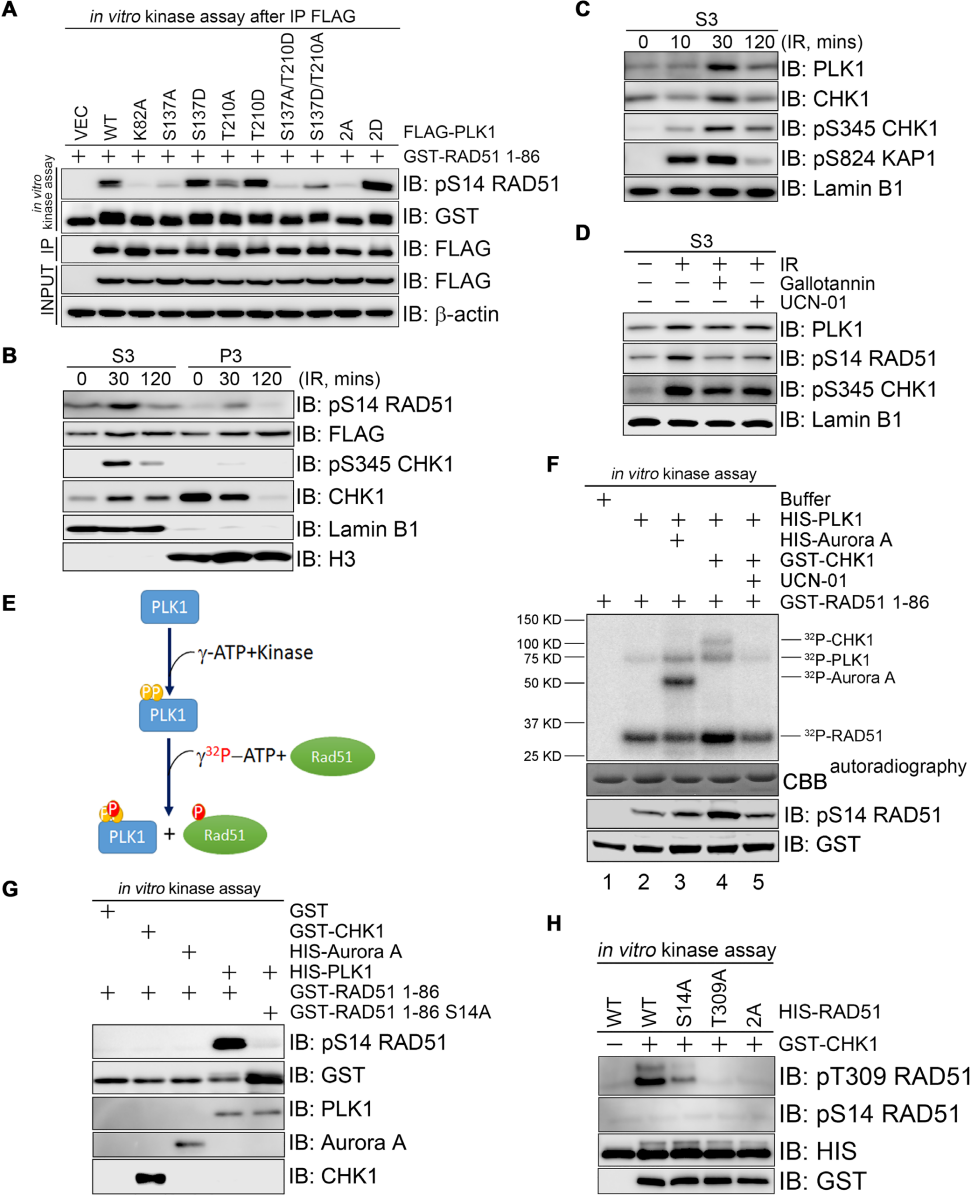
CHK1-mediated PLK1 phosphorylation at S137 promotes PLK1 enzymatic activity toward RAD51. (**A**) The effect of PLK1 S137 and/or T210 phosphorylation status on RAD51 phosphorylation. PLK1 S137 and T210 phosphorylation mutants were immunoprecipitated from 293T cells transiently expressing the indicated constructs before analysis by *in vitro* kinase assay using GST-RAD51 (1–86 aa) as a substrate. (**B**) pS345 CHK1 and pS14 RAD51 nucleoplasm levels after DNA damage. 293T cells stably expressing FLAG-RAD51 were exposed to X-ray radiation (10 Gy) at different time points and then subjected to chromatin fractionation. The nucleoplasm (S3) and chromatin-enriched (P3) fractions were analyzed by immunoblotting. (**C**) PLK1 and CHK1 protein levels in the nucleoplasm 30 min after X-ray radiation, as described in B. (**D**) pS345 CHK1 and pS14 RAD51 levels in the nucleoplasm in response to DNA damage. 293T cells stably expressing FLAG-RAD51was pretreated with gallotannin (10 μM) or UCN-01 (10 nM) for 1 h followed by 10-Gy X-ray radiation. Nucleoplasm fraction was fractionated 30 min after IR and immunoblotted with antibodies as indicated. (E, F) The effects of CHK1 and Aurora-A on PLK1 activation and subsequent RAD51 phosphorylation at S14. Schematic of the sequential kinase assay (**E**). HIS-PLK1 was phosphorylated by GST-CHK1 or HIS-Aurora A in the presence of γ-ATP before the addition of ^32^P-γ-ATP and GST-RAD51 (1–86 aa). Phosphorylation was detected by autoradiography and immunoblotting (**F**). (**G**) The effects of PLK1, CHK1 and Aurora A on direct RAD51 phosphorylation at S14. GST, GST-CHK1, HIS-Aurora A and HIS-PLK1 were incubated with GST-RAD51 (1–86 aa) or GST-RAD51 (1–86 aa) (S14A) in the presence of γ-ATP, before analysis by immunoblotting. (**H**) The effect of RAD51 phosphorylation at S14 on CHK1-mediated RAD51 phosphorylation at T309. The indicated full length RAD51 peptides were phosphorylated by GST-CHK1 in the presence of γ-ATP, before analysis by immunoblotting.

PLK1 phosphorylates RAD51 at S14 and promotes HR repair (15). We thus intended to determine the impact of the PLK1 phosphorylation status at S137 and/or T210 toward RAD51 phosphorylation. Here, wild type FLAG-PLK1 fully phosphorylated RAD51 at S14, whereas a kinase-dead mutant FLAG-PLK1 (K82A) failed to do so (Figure 6A). The phosphorylation-defective S137 mutants also exhibited minimal kinase activity toward RAD51 while the phosphorylation-mimic S137 mutants exhibited higher kinase activity compared to that of FLAG-PLK1 (Figure 6A). The FLAG-PLK1 (S137D/T210A) mutant exhibited reduced kinase activity. The phosphorylation-defective FLAG-PLK1 (T210A) mutant exhibited reduced kinase activity, whereas the phosphorylation mimic FLAG-PLK1 (T210D) mutant exhibited similar kinase activity to FLAG-PLK1 (S137D) and slightly higher activity than FLAG-PLK1. Phosphorylation at S137 is thus a prerequisite for PLK1 enzymatic activity toward RAD51.

DNA damage-activated CHK1 quickly dissociates from chromatin into the nucleoplasm (39). BRCA2 directly binds PLK1 and acts as a molecular platform to facilitate RAD51 phosphorylation at S14 in the nucleoplasm (16,24). How PLK1 is activated in the nuclear fraction is still largely unknown. We reasoned that CHK1-mediated PLK1 phosphorylation at S137 could promote PLK1 enzymatic activity toward RAD51 in the nucleoplasm. Chromatin fractionation assays confirmed that CPT treatment or IR induced a decrease in CHK1 levels in the chromatin-enriched fraction (Supplementary Figure S6, P3) and an increase in CHK1 and pS345 CHK1 in the nucleoplasm (Supplementary Figures S6, S3). Time-course experiments revealed that PLK1 and pS14 RAD51 protein levels increased within 30 min after IR and returned back to basal levels 120 min after treatment (Figure 6B and C); this increase was diminished when the cells were pretreated with gallobannin or UCN-01 (Figure 6D). This phosphorylation dynamic is consistent with PLK1 phosphorylation at S137 (Figure 4I, J). We posited that gallotannin treatment might, therefore, prevent the release of PLK1 from the PAR polymers surrounding the DNA lesion and thus reduce the level of RAD51 activation.

Because Aurora A phosphorylates PLK1 at T210 and leads to its activation (40,41), we checked whether Aurora A and/or CHK1-mediated PLK1 activation is important for RAD51 phosphorylation. Sequential *in vitro* kinase assays (Figure 6E) showed that PLK1 phosphorylated by CHK1, but not Aurora A, specifically phosphorylated RAD51 (1–86 aa) (Figure 6F, lane 4 versus lane 3) and RAD51 at S14 (Figure 6F). It was noted that Aurora A-mediated activation of PLK1 was not optimal in this assay without Bora. Nevertheless, these data demonstrated that CHK1-PLK1 axis directly targets RAD51 for phosphorylation at S14 (Figure 6G). Indeed, the surrounding sequence of S14 in RAD51 does not align with the consensus phosphorylation motifs by CHK1 (42) and Auroa A (43).

Given that RAD51 is phosphorylated at T309 by CHK1 to promote HR repair (44), we sought to clarify the relationship between RAD51 S14 and T309 phosphorylation. *In vitro* kinase assays revealed that GST-CHK1 directly phosphorylated HIS-RAD51 at T309, but not S14. This phosphorylation event was diminished when using HIS-RAD51 (S14A) as a substrate and completely abolished when using HIS-RAD51 (T309A) or HIS-RAD51 (S14A/T309A) as substrates (Figure 6H). These findings show that PLK1-mediated RAD51 phosphorylation at S14 ensures the full phosphorylation of RAD51 at T309 by CHK1.

### The CHK1–PLK1–RAD51 axis modulates DSB repair by targeting RAD51

**Figure 7.**
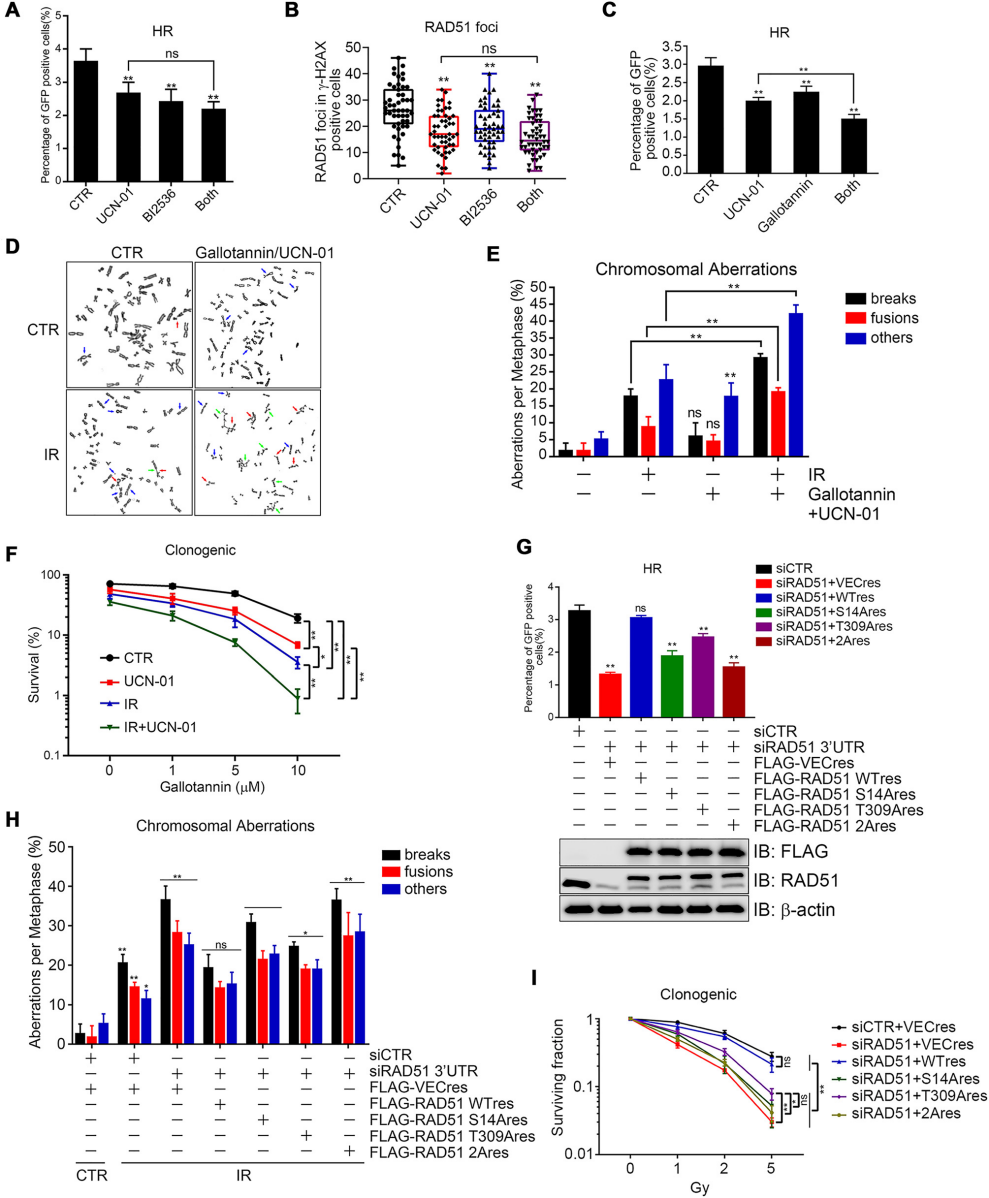
The CHK1-PLK1 axis modulates DSB repair by targeting RAD51. (**A**) The role of CHK1 and PLK1 on HR. DR-U2OS cells were infected with I-SceI for 36 h, before treatment with UCN-01 (10 nM), BI2536 (10 nM) or both for 12 h. The percentage of GFP- positive cells is shown. (**B**) The role of CHK1 and PLK1 on RAD51 focus formation. U2OS cells were synchronized at late S phase, pretreated with UCN-01 (10 nM), BI2536 (10 nM), or both for 2 h, followed by CPT (2 μM) treatment for 1 h and immunofluorescence to detect RAD51 foci in γ-H2AX-positive cells. (**C**) The effect of combined gallotannin and UCN-01 treatment on HR repair. DR-U2OS cells were infected with I-SceI for 36 h, then treated with UCN-01 (10 nM), gallotannin (10 μM), or both. The percentage of GFP-positive cells is shown. (**D**) The effect of combined gallotannin and UCN-01 treatment on chromosome aberrations. HeLa cells were pretreated with UCN-01 (10 nM), gallotannin (10 μM), or both for 12 h, X-ray irradiated (5 Gy) and then treated with colchicines (0.4 μg/ml) for 6 h before analysis by chromosome spread assay. Representative images are shown. Blue arrows (others), dicentric, deletion, ring; green arrows, fusions; red arrows, breaks. (**E**) The percentage of chromosome aberrations in part D. More than 100 mitotic chromosomes were randomly analyzed. (**F**) The effect of combined gallotannin and UCN-01 treatment on sensitizing HeLa cells to IR treatment. HeLa cells were treated with UCN-01(10 nM), X-ray irradiation (2 Gy) or both, in the presence of an increasing amount of gallotannin (0, 1, 5, 10 μM) for 2 weeks. (**G**) The effects of inhibiting RAD51 expression on HR-mediated DSB repair in U2OS cells. DR-U2OS cells stably expressing the indicated constructs were first infected with I-SceI before siRNA transfection against RAD51 (3′UTR). The repair efficiency was analyzed as in part A. (**H**) Quantitation of chromosome aberrations upon RAD51 down regulation in HeLa cells. HeLa cells stably expressing the indicated constructs or RAD51 siRNA (3′UTR) were irradiated (5 Gy) before analysis by chromosome spread assay as in part D. More than 100 mitotic chromosomes were randomly analyzed. (**I**) Relative cell survival in HeLa cells. HeLa cells stably expressing the indicated constructs were treated with RAD51 siRNA (3′UTR), irradiated (0, 1, 2, 5 Gy) and the cell survival was determined. All data are derived from three independent experiments. *P < 0.05; ** *P* < 0.01; ns, not significant.

In our final assays, we explored the biological significance of the CHK1–PLK1–RAD51 axis in the DNA damage response. We first determined if the CHK1–PLK1–RAD51 axis modulated HR-mediated DSB repair. Treating DR-U2OS cells with UCN-01, the PLK inhibitor BI2536, or both significantly reduced HR-mediated DSB repair efficiency without synergistic effects (Figure 7A). We then synchronized U2OS cells at early S phase, released them for 2 h and treated them with UCN01, BI2536 or both and CPT for 2 and 1 h, respectively. The inhibitors alone and combined significantly reduced the percentage of RAD51 foci in γ-H2AX-positive cells compared to untreated cells. Thus, CHK1 and PLK1 work in the same epistatic pathway to regulate RAD51 loading onto ssDNA during late S and G2 phases (Figure 7B, Supplementary Figure S7A and S7B). When we used the PARG inhibitor gallotannin, gallotannin alone significantly (albeit slightly) reduced HR repair efficiency. We saw an additive effect when combining gallotannin with UCN01 (Figure 7C). Given that UCN-01, BI2536 and gallotannin are very toxic to the cycling cells, we sought to determine the impact of 12 h inhibitor treatment on cell viability and cell cycle progression. We found that UCN-01, gallotannin or gallotannin plus UCN-01 treatment for 12 h did not obviously alter the cell cycle profiles by flow cytometric analysis (Supplementary Figure S7D), though, as expected, BI or BI2536 plus UCN-01 treatment resulted in slight mitotic arrest (Supplementary Figure S7D). These treatments for 12 h did not have a significant impact on cell viability by MTS assay (Supplementary Figure S7C) and percentage of cells in S phase by EdU-PI dual labeling technique (Supplementary Figure S7E and S7F) either. We then determined if ectopic expression of PLK1(S137D) mutant has a dominant-negative effect of UCN-01, B12536 and gallotannin on HR. We found that, in DR-U2OS cells stably expressing FLAG-PLK1(S137D), UCN-01 or gallotannin treatment failed to suppressed HR repair efficiency, while BI2536 treatment remained to suppress HR repair (Supplementary Figure S7G). These results suggest that ectopic expression of S137D could overwrite the negative effect of CHK1 inhibitor UCN-01 and PARG inhibitor gallotannin on HR repair, while the PLK1 inhibitor BI2536 still suppresses HR repair.

Mitotic spread and clonogenic survival assays confirmed these findings when using X-ray irradiation to induce chromosome aberrations (Figures 7D-F). Given that about 80% of IR-induced DSBs are repaired by NHEJ, we performed similar mitotic chromosome spread assays and obtained similar results (Supplementary Figure S7H and S7I), indicating defective HR functionality mainly contributes to UCN-01/gallotannin-induced chromosome aberrations.

We also found that inhibiting RAD51 expression compromised HR-mediated DSB repair, and re-expressing wild-type RAD51 rescued this defect. Re-expressing RAD51 (2A), (S14A) or (T309A) only partially rescued this defect (Figure 7G). We then determined if the CHK1–PLK1–RAD51 axis impacted on chromosome stability. Inhibiting RAD51 expression increased IR-induced chromosome aberrations, and re-expressing wild-type RAD51 rescued this defect. Re-expressing RAD51 (S14A) or (T309A) partially rescued this defect, while re-expressing (2A) did not (Figure 7H, Supplementary Figure S7J and S7K).

Finally, we determined if the CHK1–PLK1–RAD51 axis impacted on cellular radiosensitivity. Cell survival assays revealed that inhibiting RAD51 expression sensitized HeLa cells to X-ray irradiation, and this sensitization was almost fully rescued upon re-expressing an siRNA-resistant form of wild-type RAD51. Sensitization was partially rescued by re-expressing RAD51 (S14A), but not (T309A) or (2A) (Figure 7I and Supplementary Figure S7L). These findings support that the CHK1–PLK1 axis promotes DSB repair and chromosome integrity by targeting RAD51.

## DISCUSSION

We propose a molecular cascade by which PARP1 and CHK1 coordinate PLK1 enzymatic activity to promote HR repair (Supplementary Figure S8). In response to SSBs and DSBs, PARP1 is quickly recruited to the DNA damage site and PARylates early DDR factors. PARP1 synthesizes a large amount of PAR that is bound to the termini of DNA breaks and target proteins, including PARP1 itself (6). The resulting ‘PAR forest’ promotes chromatin remodeling and histone eviction and serves as a platform for recruiting DNA damage signaling and repair factors via the PAR-binding motif. The newly recruited DDR factors by the PAR forest include MRE11, CHK1 (4,7), and PLK1 (this study). Within minutes after DNA damage, PARG-mediated PAR degradation releases the PLK1–PAR complex into the nucleoplasm, allowing CHK1 to phosphorylate PLK1 at S137, then subsequently at T210 to promote PLK1’s enzymatic activity toward RAD51 at S14. PLK1-mediated RAD51 phosphorylation at S14 is a prerequisite for CHK1-mediated RAD51 phosphorylation at T309, which ultimately promotes HR-mediated DSB repair (Supplementary Figure S8). Though activated CHK1 was readily detected in the nuclear soluble fraction while barely detectable in the chromatin-enriched fraction (Figure 6B and C), we could not exclude the possibility that PAR-bound CHK1 phosphorylates PLK1 in the PAR forest, which then releases PLK1 and triggers down-stream events.

PAR binding can have inhibitory or activatory effects on target proteins. For example, DNA damage-induced PAR binding inhibits MRE11 nuclease activity (4) and blocks Aurora B kinase activity (9). Conversely, DNA damage-induced PARylation facilitates ATM activation (45), while replication stress-induced PAR binding to CHK1 enhances CHK1 activation (7). We have uncovered a novel DDR factor that is recruited by the PAR forest: PLK1. PLK1 is instantly recruited to the DSB site in a PARP-1-dependent manner by non-covalently binding to PAR via its nucleotide-binding domain. We propose that this binding efficiently inhibits its kinase activity, presumably by excluding ATP from binding to the pocket.

It was reported that PAR binding to CHK1 at stalled replication forks promotes activation of the S-phase checkpoint and cell survival in response to replication stress (7). Though not directly tested, our results indicate that DNA damage in S-phase cells could induce PAR binding to and activation of CHK1 enzymatic activity, facilitating S-phase checkpoint activation. Furthermore, activated CHK1 phosphorylates and activates PLK1 to phosphorylate RAD51 at S14, subsequentially promoting RAD51 phosphorylation at T309 directly by CHK1. These phosphorylation events fully activate RAD51 and promote HR repair. Thus, the PAR-CHK1-PLK1 axis exhibits a novel branch for mechanisms of PARylation in regulation of cell cycle checkpoint and DSB repair.

DNA damage disrupts the interaction between Aurora A and PLK1–Bora and thus inhibits PLK1 enzymatic activity in G2 phase based on the finding that expression of a fusion of Aurora A-Bora sustains PLK1 phosphorylation at T210 (35). Our data support that the CHK1–PLK1 axis, probably not the Aurora A–PLK1 axis, targets RAD51 S14 phosphorylation (Figure 6F, lanes 3 versus 4) and that CHK1-mediated PLK1 phosphorylation at S137 is superior to T210 phosphorylation in terms of PAR binding capacity (Figure 5E and H). This effect ensures PLK1 enzymatic activity toward RAD51 S14 phosphorylation (Figure 6A). Our results thus seem to support that the CHK1–PLK1 axis targets RAD51 activation in response to DNA damage during S-G2 phase of the cell cycle, while it has been reported that the CHK1-PLK1 axis regulates mitotic progress under unperturbed conditions (46,47).

A surge in PLK1-mediated RAD51 phosphorylation at S14 in response to DNA damage is thought to facilitate HR repair (15,24). In brief, pS14 RAD51 is first phosphorylated in the soluble nuclear fraction by PLK1, and then accumulates on chromatin in its pS14 and pT13/pS14 forms (16). But how PLK1 was activated in nucleoplasm was still largely unknown. We have addressed this knowledge gap, showing that CHK1-mediated PLK1 phosphorylation at S137 excludes PAR from the PAR–PLK1 complex in the nucleoplasm and promotes PLK1 activity towards RAD51 S14. The question remained, however, as to how CHK1 is activated. We previously reported that in response to replication stress, PAR binds to CHK1 through its N-terminal PAR-binding regulatory motif, promoting CHK1 activation (7). This mechanism is now supported by the following new observations: (1) CHK1 and PLK1 protein levels in the nucleoplasm increased 30 min after IR (Figure 6C); (2) CHK1 phosphorylation levels (S345) coordinately spiked 30 min after IR in the nucleoplasm (Figure 6B); (3) CHK1 phosphorylation was inhibited when cells were pretreated with gallotannin, a PARG inhibitor (Figure 6D). This latter finding indicates that PARG compromises the release of activated CHK1 from the chromatin into the nucleoplasm.

It has been debated whether PLK1 phosphorylation at S137 is essential for T210 phosphorylation and its subsequent kinase activity in the absence of a kinase for S137 phosphorylation. It has been demonstrated that S137D increases PLK1 enzymatic activity, while this phosphorylation is less abundant compared to T210 phosphorylation in mitosis under unperturbed conditions (48). We report that CHK1 preferentially targets PLK1 S137 in response to DDR, while Aurora A preferentially targets at PLK1 T210. PLK1 phosphorylation at S137 ensures optimal phosphorylation at T210 and PLK1 activation. For example, an S137 phosphorylation-defective mutant showed almost no kinase activity regardless of the T210 phosphorylation status (Figure 6A), while PARG inhibitor Gallotannin treatment-induced PAR accumulation in vivo may prevent PLK1 phosphorylation at S137, resulting in diminished phosphorylation of T210 and its substrate RAD51 (Figure 3H). Furthermore, PLK1 (S137D) exhibited higher T210 phosphorylation (Figure 4H) and higher kinase activity than PLK1 toward its artificial substrate casein (38) and physiologically relevant substrate RAD51 (S14) (Figure 6A). Meanwhile, PLK1 (S137D/T210A) exhibited moderate kinase activity (Figure 6A). These findings argue that PLK1 S137 phosphorylation is a prerequisite for optimal phosphorylation at T210 and the activation of its kinase.

PARP inhibitors (such as olaparib) effectively kill cancer cells defective in HR repair through synthetic lethality (34). PARP inhibitors might cause an increase in single strand breaks, which are converted during replication to irreparable DSBs in HR-defective cells (34). PARP inhibitors, independent of its enzymatic inhibition, also trap PARP1/2 on DNA to from stable and very cytotoxic PARP1/2-DNA complexes (49). The function of the DNA damage-induced PAR forest provides an additional explanation for the synthetic lethality exhibited by PARP1 inhibitors in HR-deficient cells. This effect could occur by a failure of: (1) activation and/or inactivation of certain DDR factors, such as PLK1; or (2) recruitment and loading of certain DDR factors into the DNA lesions such as MRE11, both of which are essential for ensuring the G2/M checkpoint and potentiating HR deficiency in S/G2 phase. This effect could also serve as the biological foundation for the combination of PARP1 and CHK1 inhibitors in ovarian cancer (50) and head and neck carcinoma (51). We reason that PARG and CHK1 inhibitors should also cause synthetic lethality: PARG inhibition sustains PLK1 inhibition at the PAR forest, while CHK1 inhibition blocks PLK1 reactivation and RAD51 phosphorylation at S14 and T309. Indeed, the PARG inhibitor gallotannin synergized with the CHK1 inhibitor UCN-01 to sensitize cancer cells to IR. This finding is consistent with a recent report that combining gallotannin and UCN-01 results in synthetic lethality in ovarian cancer cells (52). We thus propose that combined PARG inhibitor and CHK1 inhibitor therapy might serve as a novel therapeutic strategy in cancer.

## DATA AVAILABILITY

All data generated or analysed during this study are included in this published article and its supplementary data files.

## Supplementary Material

gkab584_Supplemental_File
