# Supplementary material for: PARP1 and CHK1 coordinate PLK1 enzymatic activity during the DNA damage response to promote homologous recombination-mediated repair
Source: Nucleic Acids Res. 2021 Jul 1;49(13):7554–70. doi: 10.1093/nar/gkab584 (PMC8287952; doi:10.1093/nar/gkab584)
Supplement: gkab584_Supplemental_File [file gkab584_supplemental_file.pdf]

PARP1 and CHK1 coordinate PLK1 enzymatic activity during the DNA damage response to promote homologous recombination-mediated repair

Bin Peng[1,*], Ruifeng Shi[1, 2], Jing Bian [1], Yuwei Li[1], Peipei Wang[1],Hailong Wang[3], Ji Liao[1], Wei-Guo Zhu[1], Xingzhi Xu[1,2,*]

[1]Guangdong Key Laboratory for Genome Stability & Disease Prevention and Carson International Cancer Center, Marshall Laboratory of Biomedical Engineering, Shenzhen University School of Medicine, Shenzhen, Guangdong 518060, China; [2]Shenzhen University-Friedrich Schiller Universität Jena Joint PhD Program in Biomedical Sciences, Shenzhen University School of Medicine, Shenzhen, Guangdong 518060, China. [3]Beijing Key Laboratory of DNA Damage Response and College of Life Sciences, Capital Normal University, Beijing100048, China

**Corresponding author:*

Bin Peng, PhD, Department of Cell Biology, Shenzhen University School of Medicine, Shenzhen, Guangdong 518060, China. Tel: 86-755-86930275; Email: binpeng@szu.edu.cn

Xingzhi (Xavier) Xu, PhD, Shenzhen University School of Medicine, Shenzhen, Guangdong 518060, China. Tel: 86-755-86930275; Email: Xingzhi.Xu@szu.edu.cn

Running title: PLK1 in the DNA damage response

Supplementary figures and legends

Peng et al. Figure S1

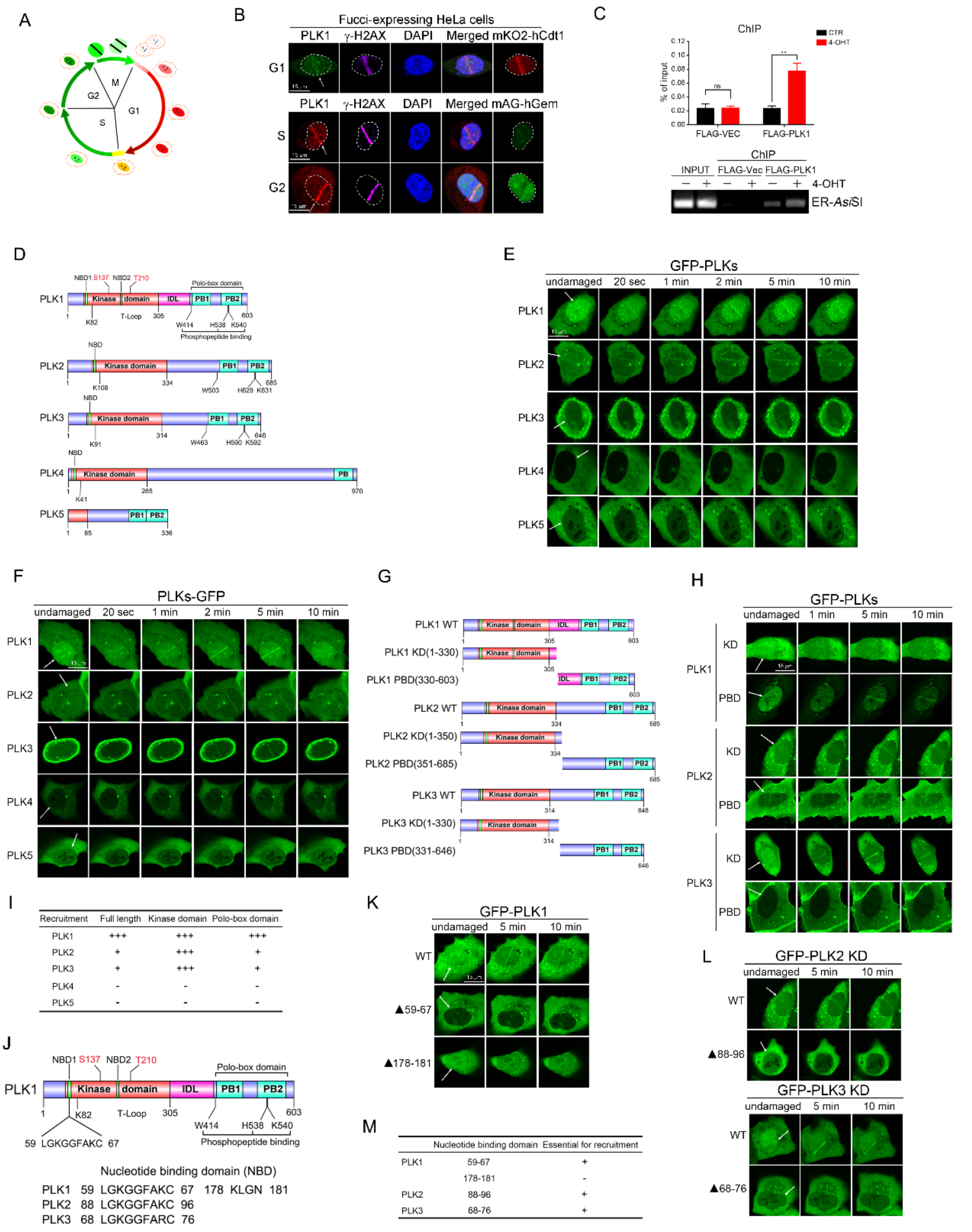

Figure S1. Recruitment of polo-like kinases to the UV laser-induced stripes

(A) Schematic representation of Fucci cell-cycle labeling. A fluorescent probe that labels individual G1 phase nuclei in red (mKO2-hCdt1) and S/G2/M phase nuclei green (mAG-hGeminin).

(B) PLK1 recruitment was correlative with its expression. Fucci HeLa cells expressing Cdt1-mKO2 only or expressing Geminin-mAG1 only were subjected to UV laser-microirradiation treatment before PLK1 and γ-H2AX analysis by immunofluorescence.

(C) Epitope-tagged PLK1 enrichment at DSB sites induced by 4-OHT. ER-*Asi*SI U2OS cells transiently expressing FLAG-VEC or FLAG-PLK1 were treated or not with 4-OHT (300 nM) for 4 h, and then analyzed by ChIP using a FLAG antibody. The data are derived from three independent experiments and represent the means ± SD. **$p < 0.01$; ns, not significant.

(D) Structural and domain features of polo-like kinases (PLKs) family. All PLK share similar architecture, with the N-terminus containing the catalytic kinase domain (KD), while the C-terminus contains polo-box domains (PBD). PLK1-4 share very similar catalytic kinase domain, while PLK5 contains a pseudo kinase domain. PLK1-3 and PLK5 possess two PBDs in tandem, while PLK4 contains a single carboxy-terminal PBD (PB). (E) Time-lapse images of recruitment of GFP-PLKs to the UV-induced DNA damage stripes. U2OS cells transiently expressing GFP tagged PLK1, PLK2, PLK3, PLK4 and PLK5 were irradiated with a 365-nm UV laser beam. Images were collected every 20 seconds after irradiation. The white arrows indicate the irradiation direction.

(F) Time-lapse images of recruitment of PLKs-GFP to the UV-induced DNA damage stripes. U2OS cells transiently expressing GFP tagged (GFP at C-terminal) PLK1, PLK2, PLK3, PLK4 and PLK5 were irradiated with a 365-nm UV laser beam. Images were collected every 20 seconds after irradiation. The white arrows indicate the irradiation direction.

(G and H) Both KD and PBD of PLK1, PLK2 and PLK3 were recruited to the DNA damage stripes. Schematic representation of kinase domain (KD) and polo-box domains (PBD) in PLK1-3 (G). U2OS cells transiently expressing GFP tagged KD or PBD of PLKs were irradiated with a 365-nm UV laser beam (H).

(I) Summary of GFP-PLK full-length, KD and PBD recruitment to DNA damage stripes. "+", weak but obvious recruitment; "+++", strong recruitment; "-", no recruitment.

(J and K) Schematic representation of nucleotide binding domains in PLK1-3 (J). Time-lapse images of recruitment of GFP-PLK1 which deleted the 1st nucleotide binding domain (59-67 aa) and 2nd nucleotide binding domain (178-191 aa) (K).

(L) Time-lapse images of recruitment of GFP-PLK2 and GFP-PLK3 which deleted the 1st nucleotide domain.

(M) Summary of GFP-PLK1-3 nucleotide binding domains recruitment to DNA damage stripes. "+", essential for recruitment; "-", not essential.

Peng et al. Figure S2

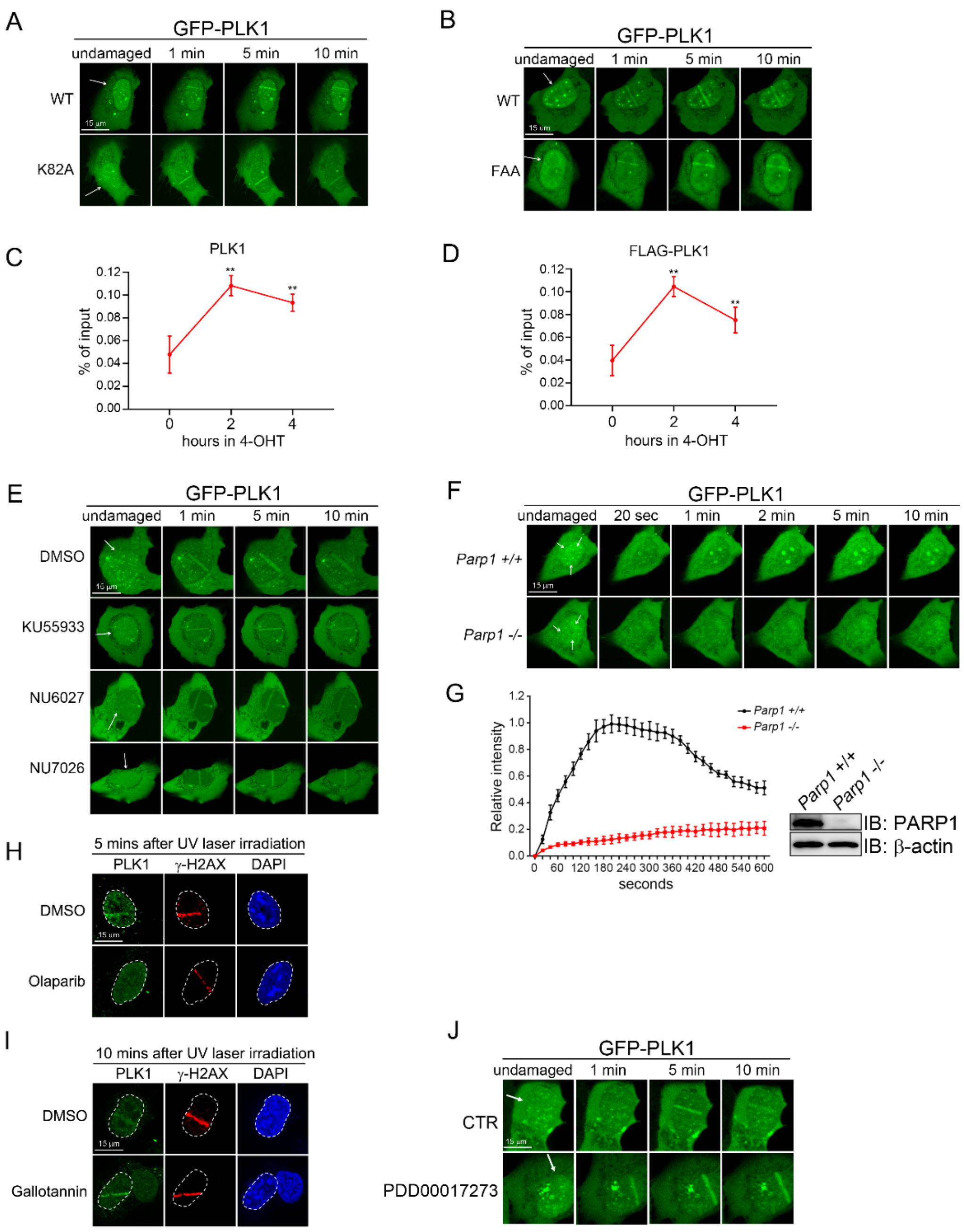

Figure S2. PLK1 recruitment to and dissociation from the DNA damage stripes are PARP-1-and PARG-dependent, respectively

(A) The requirement of PLK1 kinase activity on recruitment to DNA damage stripes. U2OS cells transiently expressing GFP-PLK1 or a catalytically inactive mutant GFP-PLK1 (K82A) were laser-microirradiated. Representative images after DNA damage are shown.

(B) The effect of FAA mutations in the polo box on PLK1 recruitment. U2OS cells transiently expressing GFP-PLK1 or GFP-PLK1 (FAA) were laser-microirradiated. Representative images after DNA damage are shown.

(C) PLK1 enrichment at ER-*Asi*S1-restriction-enzyme-induced DSBs. ChIP assay was performed in ER-*Asi*SI U2OS cells treated or not with 4-OHT (300 nM) for 2h or 4 h, using the indicated antibody. ChIP

efficiencies were measured by qPCR from *Asi*SI induced DSBs. The data represent the means ± SD. **p <0.01.

(D) Epitope-tagged PLK1 enrichment at ER-*Asi*S1-restriction-enzyme-induced DSB. ER-*Asi*SI U2OS cells transiently expressing FLAG-VEC or FLAG-PLK1 for 48 h were treated or not with 4-OHT (300 nM) for 2h or 4 h, and then analyzed by ChIP using a FLAG antibody. The data represent the means ± SD. **p <0.01.

(E) The role of ATM, ATR and DNA-PKcs in PLK1 recruitment to DNA damage sites. U2OS cells transiently expressing GFP-PLK1 were pretreated ATM (KU55933, 10 μM), ATR (NU6027, 10 μM) or DNA-PKcs (NU7026, 10 μM) inhibitors for 1 h, then laser-microirradiated. Representative images after DNA damage are shown.

(F-G) PLK1 recruitment to the DNA damage sites in *Parp-/-* MEFs. WT and *Parp-/-* MEF cells transiently expressing GFP-PLK1 were laser-microirradiated. Representative images (F) and the quantitation of the GFP-PLK1 fluorescence intensity at DNA damage sites (G) are shown.

(H) The effect of PARP1 inhibition on endogenous PLK1 recruitment to DNA damage stripes. U2OS cells were pretreated with olaparib (1 μM) for 2 h, and then laser-microirradiated. The cells were fixed 5 min after irradiation and analyzed with the indicated antibodies.

(I-J) The effect of PARG inhibition on sustaining endogenous PLK1 at DNA damage stripes. U2OS cells were pretreated with gallotannin (10 μM) or PPD00017273 (10 μM) for 2 h, then laser-microirradiated. The cells were fixed 10 min after irradiation and analyzed with the indicated antibodies.

Data in (C), (D), and (G) are derived from three independent experiments.

Peng et al. Figure S3

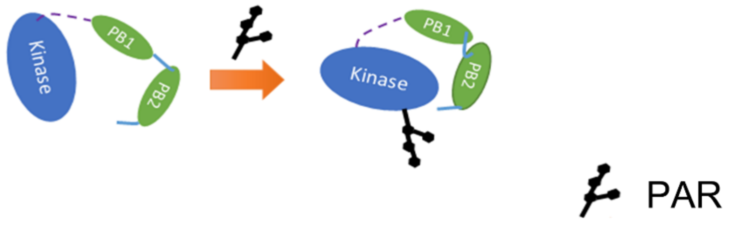

Figure S3. A proposed model explaining how PAR might promote the intramolecular interaction between the PBD and the kinase domain to suppress PLK1 kinase activity.

Peng et al. Figure S4

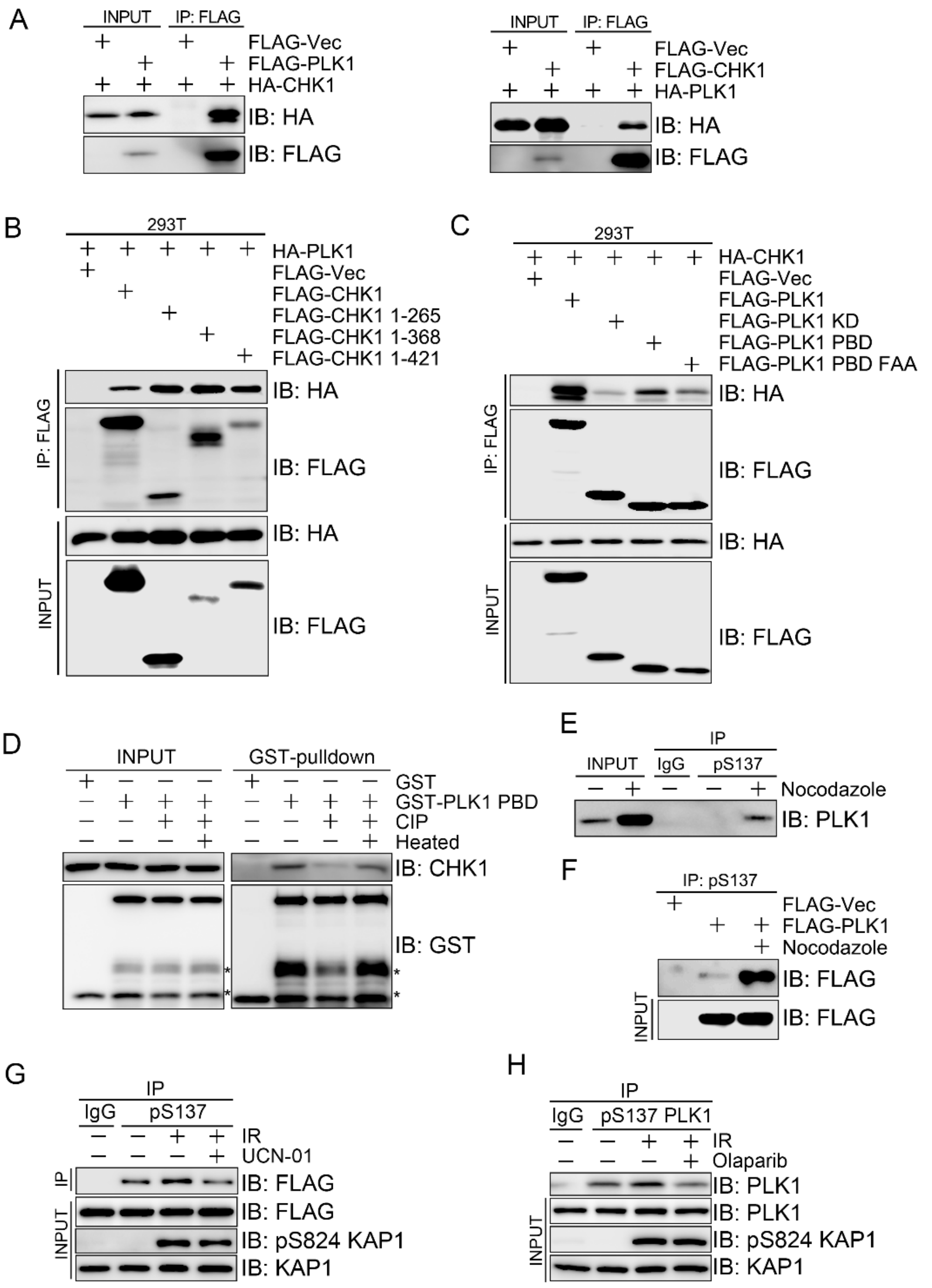

Figure S4. CHK1 phosphorylates PLK1 at S137

(A) The interaction between epitope-tagged PLK1 and CHK1. Total 293T cell lysates expressing HA-CHK1 and FLAG-VEC or FLAG-PLK1 (left panel), or HA-PLK1 and FLAG-VEC or FLAG-CHK1 (right panel) were immunoprecipitated with a FLAG antibody and analyzed by immunoblotting.

(B) The role of the CHK1 kinase domain on mediating an interaction with PLK1. Total 293T cell lysates transiently expressing HA-PLK1 and FLAG-VEC, FLAG-CHK1, FLAG-CHK1 (1-265), FLAG-CHK1 (1-368) or FLAG-CHK1 (1-421) were immunoprecipitated with a FLAG antibody and analyzed by immunoblotting.

(C) The role of the PLK1 KD and PBD in mediating the interaction with CHK1. Total 293T cell lysates expressing HA-CHK1 along with FLAG-VEC, FLAG-PLK1, FLAG-PLK1 KD, FLAG-PLK1 PBD, or

FLAG-PLK1 PBD (FAA) were immunoprecipitated with a FLAG antibody and analyzed by immunoblotting.

(D) The dependency of phosphorylation on the CHK1–PLK1 interaction. GST-PLK1 PBD was used to pulldown endogenous CHK1 from total 293T cell lysates in the presence or absence of CIP, or heat-inactivated CIP (100℃ for 10 min). Asterisk represent the degradation band of GST-PLK1 PBD.

(E)The suitability of a phospho-specific pS137 PLK1antibody for immunoprecipitating endogenous PLK1 phosphorylated at S137. Total cell lysates derived from 293T cells pretreated or not with nocodazole (340 nM) for 16 h were immunoprecipitated with the pS137 PLK1antibody and analyzed by immunoblotting with a PLK1 antibody.

(F) The suitability of a phospho-specific pS137 PLK1 antibody for immunoprecipitating FLAG-PLK1 phosphorylated at S137. 293T cells transiently expressing FLAG-VEC or FLAG-PLK1 pretreated or not with nocodazole (340 nM) for 16 h, were immunoprecipitated with the pS137 PLK1antibody and analyzed by immunoblotting.

(G) The effect of UCN-01 on the X-ray radiation-induced surge of PLK1 phosphorylation at S137. Total cell lysates derived from 293T cells pretreated with UCN-01 (10 nM) for 60 min before X-ray radiation (10 Gy), then immunoprecipitated after 30 min with a pS137 PLK1 antibody and analyzed by immunoblotting.

(H) DNA damage-induced PLK1 phosphorylation at S137 after Olaparib treatment. 293T cells were pre-treated with Olaparib (1 μM) for 60 min before X-ray radiation (10 Gy), total cell lysates derived from these cells 30 min after IR were immunoprecipitated after 30 min with pS137 PLK1 antibody followed by immunoblotting with antibodies as indicated.

Peng et al. Figure S5

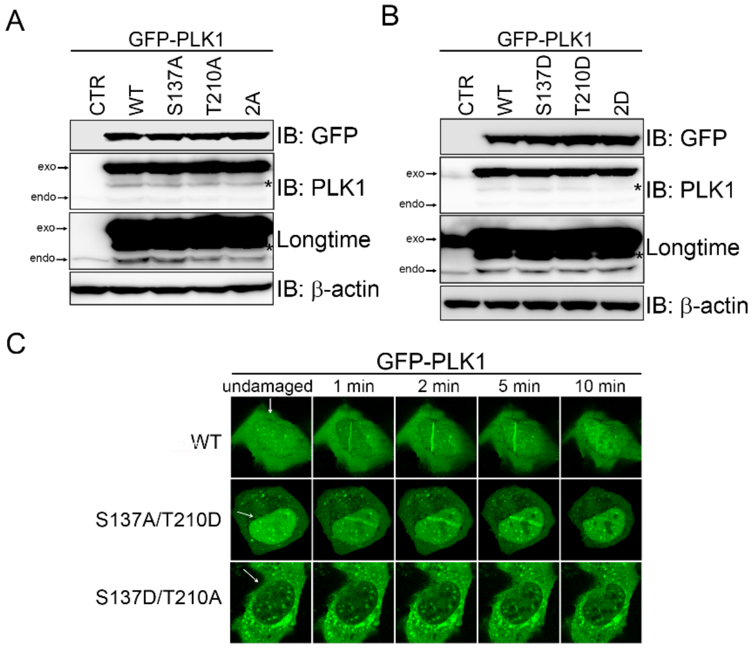

Figure S5. PLK1 (S137) phosphorylation despite of T210 phosphorylation status blocks its recruitment to the DNA damage sites

(A) U2OS cells transiently expressing GFP, GFP-PLK1 WT, S137A, T210A and 2A for 48 hours, followed by immunoblotting with antibody as indicated.

(B) U2OS cells transiently expressing GFP, GFP-PLK1 WT, S137D, T210D and 2D for 48 hours, followed by immunoblotting with antibody as indicated.

(C) U2OS cells transiently expressing the indicated constructs were laser-microirradiated. Representative images taken at the indicated time points are shown.

Peng et al. Figure S6

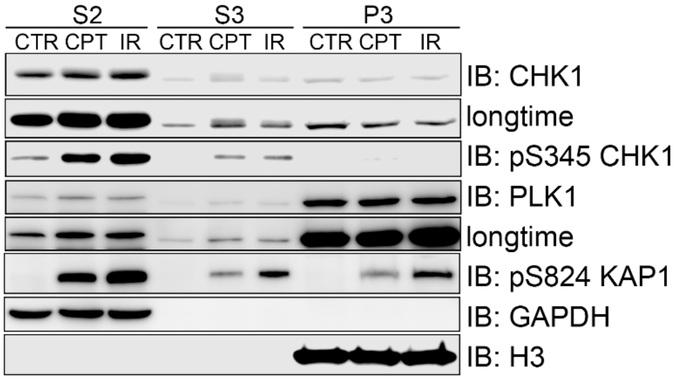

Figure S6. DNA damage induces a surge of CHK1 and PLK1 in the nucleoplasm. DNA damage induces a surge of CHK1 and PLK1 in the nucleoplasm. 293T cells treated with CPT (2 μM) for 30 min or exposed to X-ray radiation (10 Gy) for 30 min were subjected to chromatin fractionation and analyzed by immunoblotting. S2: cytoplasm fraction, S3: nucleoplasm fraction, P3: chromatin-enriched fraction.

Peng et al. Figure S7

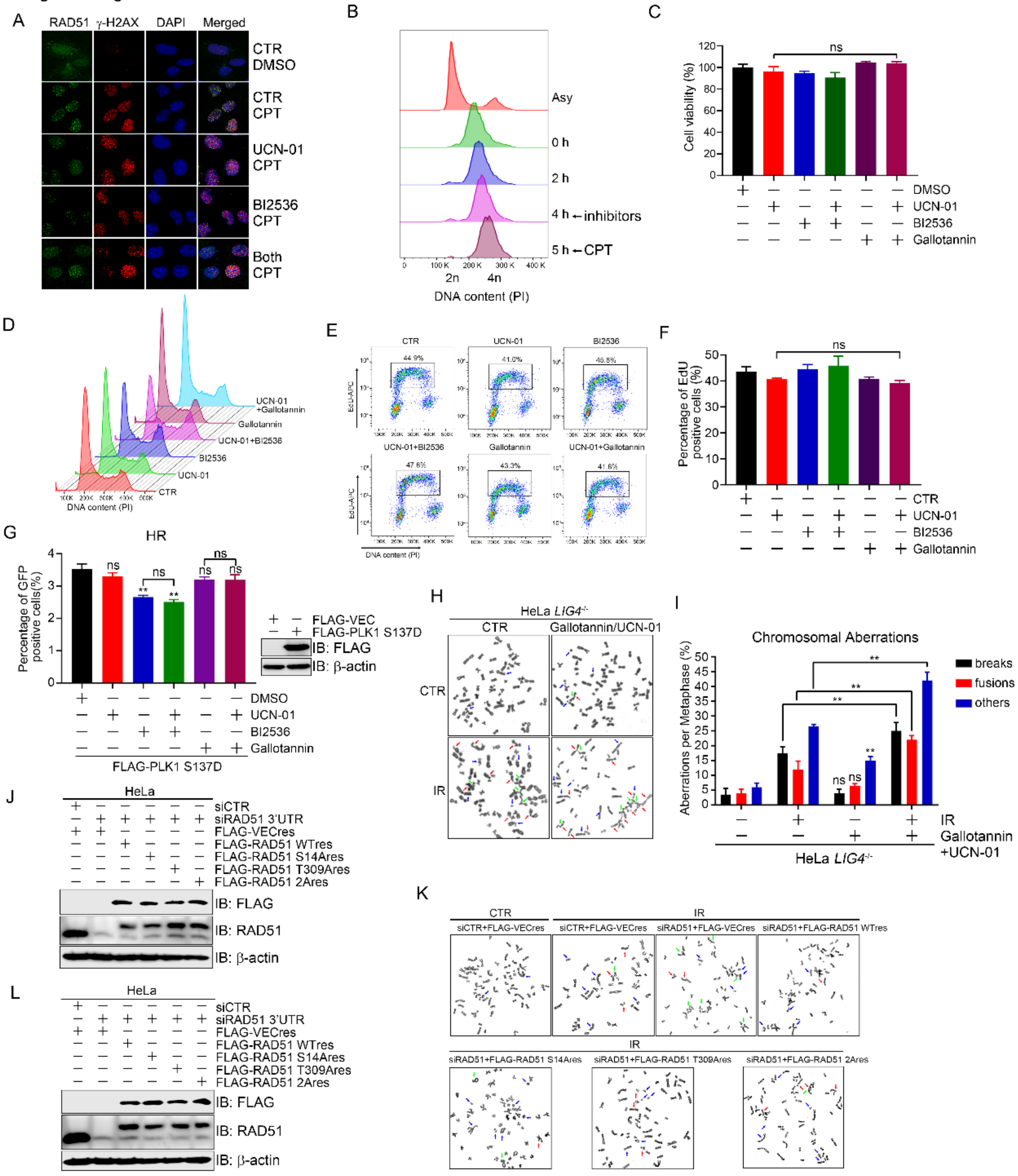

Figure S7. The CHK1-PLK1 axis modulates DSB repair by targeting RAD51

(A and B) Representative images of RAD51 focus formation and cell cycle profile analysis. U2OS cells were synchronized at early S phase by double-thymidine block, then released to progress from early S phase to late S phase. Synchronized cells released from double-thymidine block for 2 h were treated with UCN-01 (10 nM), BI2536 (10 nM), or both for 2 h, followed by CPT (2 μM) treatment for 1 h. The cells were analyzed by immunofluorescence using RAD51 and γ-H2AX antibodies (A). The cell cycle profile analysis was shown in (B).

(C) Cell viability upon inhibitor treatment. U2OS cells were treated with UCN-01 (10 nM), BI2536 (10 nM), UCN-01 plus BI2536, Gallotannin (10 μM), and Gallotannin plus UCN-01 for 12 h, followed by MTS assay to measure the relative cell viability.

(D-F) Cell cycle profile analysis upon inhibitor treatment. U2OS cells were treated with UCN-01 (10 nM), BI2536 (10 nM), UCN-01 plus BI2536, Gallotannin (10 μM) and Gallotannin plus UCN-01 for 12

h, followed by PI staining and flow cytometric analysis (D), EdU labeling for S-phase cells (E), and subsequent quantification of S phase cells (F).

(G) The role of CHK1 and PLK1 on HR. DR-U2OS cells stably expressing FLAG-PLK1 S137D were infected with I-SceI lentivirus for 36 h. Cell were pre-treated with UCN-01 (10 nM), BI2536 (10 nM), gallotannin (10 μM), UCN-01 plus BI2536, or UCN-01 plus gallotannin for 12 h before harvest. The percentage of GFP positive cells and western blotting were shown in G.

(H and I) The effect of combined gallotannin and UCN-01 treatment on chromosome aberrations. LIG4 KO HeLa cells were pretreated with UCN-01 (10 nM), gallotannin (10 μM), or both for 12 h, X-ray irradiated (5 Gy) and then treated with colchicines (0.4 μg/ml) for 6 h before analysis by chromosome spread assay. Representative images are shown. Blue arrows (others), dicentric, deletion, ring; green arrows, fusions; red arrows, breaks.

(J and K) Representative images (K) and immunoblot analysis (J) of chromosome aberrations upon RAD51 depletion or re-expression with its variants (shown in Figure 7H). RAD51 was depleted by transfecting HeLa cells stably expressing the indicated constructs with siRAD51 (3'UTR). The transfectants were X-ray radiated (5 Gy) and then exposed to colchicines (0.4 μg/ml) for 6 h before harvesting. The cells were analyzed by immunoblotting with the indicated antibodies (J). Chromosome spreads were performed and images were captured under a confocal imaging system. Blue arrows (others), dicentric, deletion, ring; green arrows, fusions; red arrows, breaks.

(L) Immunoblot analysis of the cell samples shown in Figure 7I. RAD51 was depleted by transfecting HeLa cells stably expressing the indicated constructs with siRAD51 (3'UTR). Total cell lysates were extracted 48 h after transfection and analyzed by immunoblotting.

All data are derived from three independent experiments.

Peng et al. Figure S8

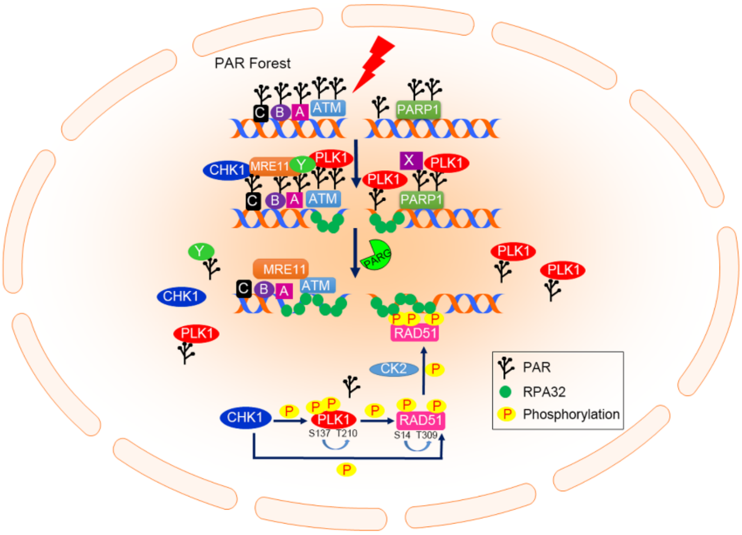

Figure S8. A working model for PARP1 and CHK1 to coordinate PLK1 kinase activity toward RAD51 during the DNA damage response. See detailed description in the Discussion. A, B, and C stand for PARylated proteins, while X and Y represent PAR-binding proteins.
